# Protein citrullination marks myelin protein aggregation and disease progression in mouse ALS models

**DOI:** 10.1186/s40478-022-01433-5

**Published:** 2022-09-08

**Authors:** Issa O. Yusuf, Tao Qiao, Sepideh Parsi, Ronak Tilvawala, Paul R. Thompson, Zuoshang Xu

**Affiliations:** 1 Department of Biochemistry and Molecular Biotechnology, University of Massachusetts Medical School, Worcester, MA 01605 USA; 2 Present Address: Astellas Pharma, 33 Locke Dr, Marlborough, MA 01752 USA; 3 Present Address: Center for Systems Biology, Massachusetts General Hospital and Harvard Medical School, Boston, MA 02114 USA; 4 Present Address: Scorpion Therapeutics, 1 Winthrop Square, Boston, MA 02110 USA; 5 Program in Chemical Biology, University of Massachusetts Medical School, Worcester, MA 01605 USA

**Keywords:** Neurodegeneration, Deimination, Neurodegenerative disease, Myelin degeneration, Astrogliosis, Protein aggregation

## Abstract

Increased protein citrullination (PC) and dysregulated protein arginine deiminase (PAD) activity have been observed in several neurodegenerative diseases. PC is a posttranslational modification catalyzed by the PADs. PC converts peptidyl-arginine to peptidyl-citrulline, thereby reducing the positive charges and altering structure and function of proteins. Of the five PADs, PAD2 is the dominant isoform in the central nervous system (CNS). Abnormal PC and PAD dysregulation are associated with numerous pathological conditions, including inflammatory diseases and neurodegeneration. Animal model studies have shown therapeutic efficacy from inhibition of PADs, thus suggesting a role of PC in pathogenesis. To determine whether PC contribute to amyotrophic lateral sclerosis (ALS), a deadly neurodegenerative disease characterized by loss of motor neurons, paralysis, and eventual death, we investigated alterations of PC and PAD2 in two different transgenic mouse models of ALS expressing human mutant SOD1^G93A^ and PFN1^C71G^, respectively. PC and PAD2 expression are altered dynamically in the spinal cord during disease progression in both models. PC and PAD2 increase progressively in astrocytes with the development of reactive astrogliosis, while decreasing in neurons. Importantly, in the spinal cord white matter, PC accumulates in protein aggregates that contain the myelin proteins PLP and MBP. PC also accumulates progressively in insoluble protein fractions during disease progression. Finally, increased PC and PAD2 expression spatially correlate with areas of the CNS with the most severe motor neuron degeneration. These results suggest that altered PC is an integral part of the neurodegenerative process and potential biomarkers for disease progression in ALS. Moreover, increased PC may contribute to disease-associated processes such as myelin protein aggregation, myelin degeneration, and astrogliosis.

**Supplementary Information:**

The online version contains supplementary material available at 10.1186/s40478-022-01433-5.

## Introduction

ALS is a devastating and fatal neurodegenerative disorder characterized by motor neuron loss, muscle wasting, and paralysis [1, 2]. Research indicates multiple mechanisms contribute to disease pathogenesis, including genetic mutations, protein aggregation, aberrant RNA processing, mitochondrial dysfunction, Endoplasmic Reticulum (ER) stress, cytoskeleton disruption, reactive oxidative species, neuroinflammation, non-cell autonomous toxicity, nuclear impairment, and white matter degeneration [3–6]. Despite significant advances in understanding the disease in recent years, the disease mechanism underlying ALS remains elusive, diagnosis of the disease remains slow and difficult, and no treatment can halt or reverse progression of the disease. Therefore, continuing effort is needed to expand our understanding of the disease.

Protein citrullination (PC), or deimination, is a widespread posttranslational modification (PTM) that is catalyzed by the protein arginine deiminases (PADs) and converts peptidyl-arginine to peptidyl-citrulline [7, 8]. The reaction removes positive charges from proteins, thereby altering protein structure and functions that include activity and interactions with other proteins and nucleic acids [9–12]. Mammals express five PADs: PADs 1–4, and PAD 6. PADs 1–4 are enzymatically active whereas PAD 6 is inactive. Activity of PADs is tightly regulated by calcium levels [13]. Under normal cellular calcium concentrations (~ 100 nM), PADs are inactive. However, they are activated at high calcium levels (1–10 mM). Once activated, PADs citrullinate a wide range of protein substrates thus regulating various cellular processes including cell signaling, immune responses, gene regulation, and myelination [14]. PADs are differentially expressed in tissues, and they have common as well as distinct substrate specificities [7]. In the CNS, PAD2 is the most dominantly expressed PAD [15].

PADs and citrullination are involved in many pathological conditions, such as rheumatoid arthritis, atherosclerosis, lupus, COVID-19 and cancers [16, 17]. Dysregulated PAD2 activity and PC are also observed in neurodegenerative conditions, including Alzheimer’s disease (AD), Parkinson’s disease (PD), prion disease, multiple sclerosis (MS), and ischemic and traumatic brain injuries [18–26]. PAD inhibitors have shown remarkable efficacy in models of several diseases, including tissue inflammation, autoimmune diseases, CNS injuries, and MS [22, 27–30]. Furthermore, the presence of anti-PAD2 antibodies is associated with less severe disease in human MS [31]. These results suggest that dysregulated PC contributes to pathological conditions and may be harnessed for diagnosis and targets for therapy.

Given that PC and PAD alterations have been linked to neurodegenerative diseases, and the potential for targeting PADs and PC for therapy and diagnosis, we investigated alterations in PC and PAD expression in two ALS mouse models expressing human mutant SOD1^G93A^ and PFN1^C71G^, respectively. We show that PC and PAD2 expression progressively increase as pathogenesis develops in the spinal cord of both ALS models. These increases coincide with the CNS areas that show the most severe motor neuron degeneration, including spinal cord and brainstem. At the cellular level, PC and PAD2 accumulate prominently in astrocytes, increasing in parallel with the accumulation of GFAP, a widely used astrogliosis marker. While PC and PAD2 accumulate in astrocytes, they are decreased in neurons. In spinal cord white matter, citrullinated proteins accumulated in protein aggregates. These aggregates are mostly colocalized with the myelin proteins proteolipid protein (PLP) and myelin basic protein (MBP), and to a much lesser degree, with disease-specific mutant protein aggregates formed by mutant SOD1 and PFN1. Furthermore, citrullinated proteins were overwhelmingly enriched in insoluble protein fractions. These results indicate that increased PC and PAD2 dysregulation are pathological hallmarks of ALS along with reactive astrogliosis, axonal degeneration, and protein aggregation, and suggest that the dysregulation of PAD2 and PC contribute to ALS pathogenesis.

## Material and methods

### Transgenic mice

Transgenic mice with high expression of human mutant SOD1^G93A^ (B6SJL-Tg(SOD1*G93A)1Gur/J, stock No. 002726) and human wild type SOD1 (B6SJL-Tg(SOD1)2Gur/J, stock No. 002297) were purchased from Jackson Lab (Bar Harbor, ME), and bred onto a FVB/NJ background for more than ten generations. Genotyping was conducted according to the protocol from the Jackson Lab. Transgenic mice expressing human mutant PFN1^C71G^ and human wild type PFN1 were generated on an FVB/NJ background, genotyped, and maintained as previously reported [32]. PFN1^C71G^ and PFN1^WT^-expressing mice are available from the Jackson Lab as FVB-Tg(Prnp-PFN1*C71G)#Zxu Tg(THY1-PFN1*C71G)67#Zxu/J, stock No. 028608, and FVB/N-Tg(Prnp-PFN1)34Zxu/J, stock No. 028607, respectively. Genotyping primers used for the PFN1 mice model are, forward: TTG AAA GAG CTA CAG GTG GA, reverse: GTA AGC CTA TCC CTA ACC CT; and SOD1 mice, forward: CAT CAG CCC TAA TCC ATC TG, reverse: CGC GAC TAA CAA TCA AAG TGA. Age-matched non-transgenic (nTg) FVB/NJ mice and wild-type transgenic mice were used as controls. Disease stages for PFN1^C71G^ mice were defined as follows: presymptomatic: age 13 weeks or younger. During this stage, the average grip strength increases to peak, and the in-cage behavior is indistinguishable from the nTg controls. Onset: age 14–18 weeks. During this stage, the average grip strength progressively weakens. Most mice display mild symptoms including leg tremors and not fully extending when lifted by tail, walking slightly slower than normal controls and hesitant. Progression: age 19–29 weeks. During this stage, the average grip strength continues declining. The symptoms become progressively more severe. The mice are unable to extend legs when lifted by the tail. Walking is slow with frequent and lengthening pauses. Paralysis: average 30 weeks ± 3 weeks (standard deviation), n = 45. At this stage, the mice become incapable of locomotion and cannot right themselves when placed on either side within 10 s, or any two limbs become completely immobile. Disease stages for SOD1^G93A^ mice were defined as follows: presymptomatic: age 10 weeks or younger. During this stage, the average body weight increases progressively. Onset: age 13 to 16 weeks. During this stage, the average body weight plateaus. Progression: age 17 to 19 weeks. During this stage, the average body weight declines. Paralysis: average 20 weeks ± 2 weeks (standard deviation), n = 18. During this stage, the average body weight decreases dramatically. The symptoms of each disease stage are the same as described above for the PFN1^C71G^ mice. Mice were maintained at the University of Massachusetts Medical School animal facility and used according to the guidelines set forth by the Institutional Animal Care and Use Committee (IACUC). All mice were euthanized when they reach the paralysis stage.

### RNA isolation and quantitative real-time PCR

Total RNA was isolated from lumber spinal cord tissue (Trizol™ Reagent; ThermoFisher Scientific, 15596018) according to the manufacturer’s instructions. The isolated RNA was transcribed to cDNA using iScript cDNA Synthesis Kit (Bio-Rad, 1708891). Gene expression levels were measured by quantitative real-time PCR (RT-qPCR) using SsoFast™ EvaGreen® Supermix (Bio-Rad,1725200). CFX96™ Touch Real-Time PCR detection system was used with the following protocol: 2 min of pre-denaturation at 95 °C, 45 consecutive cycles of 5 s denaturation at 95 °C, 20 s annealing at 60 °C. 60S ribosomal protein L32 (RPL-32) was used as control for the quality and quantity of cDNA preparation. The following primer pairs were used: PAD2, forward (5'-GTTATGTTCAAGGGCCTGGGAGGCATG-3'), reverse (5′-TAGCACGATCATGTTCACCATGTTAGG-3′); PAD1, forward (5′-CTTCAAGGTGAAGGTGTCATACT-3′), reverse (5′-CGACATCAAGGGACACATCAA-3′); RPL-32 forward (5′-ATGGCTCCTTCGTTGCTG C-3′), reverse (5′-CTGGACGGCTAATGCTGGT-3′).

### Western blot

Mice were deeply anesthetized and decapitated. Tissues were quickly harvested, snap-frozen in liquid N_2_, and stored at − 80 °C. For protein preparation, frozen tissues were homogenized in homogenization buffer composed of 25 mM phosphate pH 7.6, 5 mM EDTA, 1% (vol/vol) SDS, 0.5% (vol/vol) Triton X-100, 0.5% (vol/vol) deoxycholic acid, protease and phosphatase inhibitor cocktail (Thermo Scientific™, 78442) and then centrifuged at 16,060×*g* for 10 min at 4 °C. The supernatant was collected as the protein sample. Protein concentration was measured using the Bradford assay (Bio-Rad, 5000006). The samples were heated in Laemmli buffer (Bio-Rad 1610747) plus 10% β-Mercaptoethanol (Bio-Rad, 1610710) at 95 °C for 10 min, and equal amounts of protein were loaded and resolved by SDS-PAGE (Bio-Rad). Proteins were transferred to nitrocellulose membranes (Amersham™ Protran®, GE10600002). The blots were blocked with 5% (wt/vol) nonfat dry milk (Boston Bioproducts Inc., P-1400) in PBS for 1 h, incubated with primary antibodies overnight at 4 °C, washed with phosphate-buffered saline with 0.1% Tween 20 (PBST) three times for 5 min each, incubated with horseradish peroxidase (HRP)–linked secondary antibodies in PBST with 5% (wt/vol) nonfat dry milk for 1 h at room temperature (RT), and washed three times for 5 min each. Protein bands were visualized using SuperSignal™ West Pico PLUS Chemiluminescent Substrate (Thermo Scientific™, 34580) reagent and detected by the Amersham Imager 600 (GE). The integrated density of the protein bands was quantified using ImageJ software. See Additional file 1: Tables 1 and 2 for the list of primary and secondary antibodies.

### Citrullinated protein detection

Citrulline modification was detected using the anti-modified citrulline (AMC) detection kit (Millipore, 17-347B). For detection on immunoblots, proteins were resolved by SDS-PAGE and transferred to PVDF membrane (Thermo Scientific™, 88518). The membranes were washed two times with tap water and incubated with AMC reagent according to the manufacturer’s instructions. The membranes were rinsed 4–5 times with tap water, blocked in freshly prepared 5% nonfat dry milk in tris-buffered saline with 0.1% Tween 20 (TBST) for 1 h at RT, incubated with anti-modified citrulline antibody (Millipore, MABS487) diluted in freshly prepared TBST-milk (1:1000) for 2 h at RT, washed three times with TBST for 10 min each, incubated with goat anti-Human IgG HRP-conjugate in TBST-Milk (1:2000) for 1 h at RT, and washed three times with TBST for 10 min each. All incubation steps were conducted with constant agitation. Finally, the membranes were rinsed one time with water, and the staining signal was visualized as described above (Western blotting).

For immunostaining citrullinated proteins, the method of Asaga and Senshu was applied with modifications [33]. This procedure is similar to the procedure for protein blots described above except that tissue sections were treated with AMC reagent. The sections were washed with PBST three times for 10 min each, blocked for 1 h and then incubated with anti-modified citrulline antibody (1:100) overnight at RT. Following washing, the sections were incubated with secondary antibody for 2 h at RT, washed again, and mounted on slides. Images were taken with a confocal microscope (see below).

### Sedimentation assay

Mouse cervical spinal cords were homogenized using a handheld polytron for 20 s in lysis buffer [50 mM Tris–HCl, 150 mM NaCl, 0.5% deoxycholic acid, 1% (v/v) Triton X-100, 5 mM EDTA] with protease and phosphatase inhibitor cocktail (1:100 dilution, Thermo Scientific™, 78442). The tissue to buffer ratio was 1:10 (mg/µL). The homogenates were centrifuged at 12,000×*g* at 4 °C for 5 min. The supernatants were moved to another tube and protein concentrations were determined using Bradford method. The pellets were rinsed three times with PBS, dissolved in 50 µL Laemmli buffer by heating to 95 °C for 10 min and a brief sonication (70 W, 50% output, 10–20 s), and then cleared by centrifugation. 80 µg of supernatant protein and the pellet amount equivalent to the three times of the volume of the supernatant containing the 80-µg protein from each sample were resolved by SDS-PAGE, and proteins were transferred to a PVDF membrane (Thermo Scientific™, 88518). Western blots for detecting proteins and citrullinated proteins were as described above.

### Filter trap assay

Spinal cord homogenate preparation and protein concentration measurement are described in the sedimentation assay section. 200 µg of protein were equalized with lysis buffer of the same volume, and then diluted with 20 volumes of PBS (pH 7.4) containing 1% SDS. The solution was sonicated (70 W, 50% output, 30 s) and then filtered under vacuum through a pre-wet 0.45 mm pore size PVDF membranes (Thermo Scientific™, 88518) using a 96-well dot-blot apparatus (Schleicher and Schuell, Inc.). Each well was washed two times with PBS. The membrane was removed from the apparatus and rinsed in tap water. Citrullinated proteins and other proteins were detected as described above.

### Immunofluorescence and immunohistochemistry

Mice were anesthetized and perfused transcardially with cold PBS followed by 4% paraformaldehyde (PFA) in PBS. The perfused mice were then immersed in the same fixative at 4 °C for another 24–48 h. Tissues were dissected and immersed in PBS containing 30% (wt/vol) sucrose at 4 °C for 2–3 days, then frozen in optimal cutting temperature (OCT) freezing media (Sakura) and stored at − 80 °C. Spinal cord frozen cross-sections were cut at 20 μm using a cryostat. For immunostaining, sections were treated with BLOXALL® Endogenous blocking solution (Vector Lab, SP-6000-100) for 10 min followed by 10 min wash with 1 × PBS. The sections were incubated in blocking solution [5% (vol/vol) donkey or goat serum (Sigma, D9663; Sigma, G9023), 0.15% Triton-X100 and (2%) nonfat dry milk in PBS, pH 7.4] for 1 h at RT. In the case of mouse-on-mouse staining procedure, anti-mouse IgG Fab fragments (Jackson Immuuno Research Laboratories, Inc., 115-007-003) was added to the blocking solution. Following the blocking step, the sections were immunostained as follows: incubation with a primary antibody in the blocking solution overnight at 4 °C with continuous agitation, washing three times for 10 min each and incubation with the appropriate secondary antibody at RT for 2 h. For immunofluorescence, the sections were washed three times in PBST for 10 min each and mounted on super frost plus slide (Fisher scientific, 15-188-48) using Vectashield mounting medium containing DAPI (Vector Laboratories, H-1800) and sealed with nail polish. Images of the spinal cord sections were taken with a Leica confocal microscope.

For immunohistochemistry, deparaffinated sections were washed three times in PBS containing 0.25% (v/v) Tween 20, stained following the manufacturer’s instructions for Vectastain ABC kit, Elite PK-6100 standard, then ImmPact DAB peroxidase Substrate kit (Vector Lab, SK-4105), and counterstained with Hematoxylene (Vector lab, H-3401). The sections were then mounted on slides and dried in an oven overnight at 55 °C. After soaking in Xylene twice for 2 min each, the slides were sealed with Permount mounting medium (Fisher Chemical SP15-100). Images of the spinal cord sections were taken with a Nikon Optiphot microscope equipped with a SPOT Insight 2.0 Mp Firewire Color digital camera. See Additional file 1: Tables 1 and 2 for the list of primary and secondary antibodies.

### Quantitative image analysis

The parameters of staining and imaging were identical for all images taken in each study. Multiple sections from each animal were studied. ImageJ was used to process and assess all images. For quantification of staining intensity in neurons in ventral horn spinal cord, we measured PAD2 or citrulline staining intensity from the nucleus of individual NeuN-positive cells. For quantification of GFAP and NF-L, the staining intensity was measured from individual image frames. For quantification of PAD2 and citrulline in neurons and glia, see Additional file 2: Fig. S9. For quantification of aggregates, confocal images were taken from ventral and ventrolateral spinal cord white matter. Because the aggregates are of very high staining intensity, citrulline-stained images were taken at relatively low exposure to exclude background signal and low intensity signal from neurofilaments in axons. A cut-off for aggregate size was set at 10 µm^2^. Signal from astrocytic processes was excluded by applying a shape factor filter to include only structures with shape circularity of 0.4–1. The few remaining linear astrocytic processes were eliminated manually. For quantification of aggregate colocalization with myelin proteins, Manders colocalization coefficient was determined by Just Another Colocalization Plugin (JACoP) [34, 35] in ImageJ (See Additional file 2: Fig. S10).

### Statistics

Results are shown as mean ± standard error of mean (SEM). GraphPad Prism (GraphPad Software Inc. vr. 8.4.3) was used to analyze statistical difference. Student’s *t* test was used to analyze differences in two sample comparisons. One-way ANOVA with Bonferroni post hoc test was used in comparisons of more than two groups. Statistical significance was set at *p* < 0.05.

## Results

### PC changes dynamically during the disease progression

**Fig. 1:**
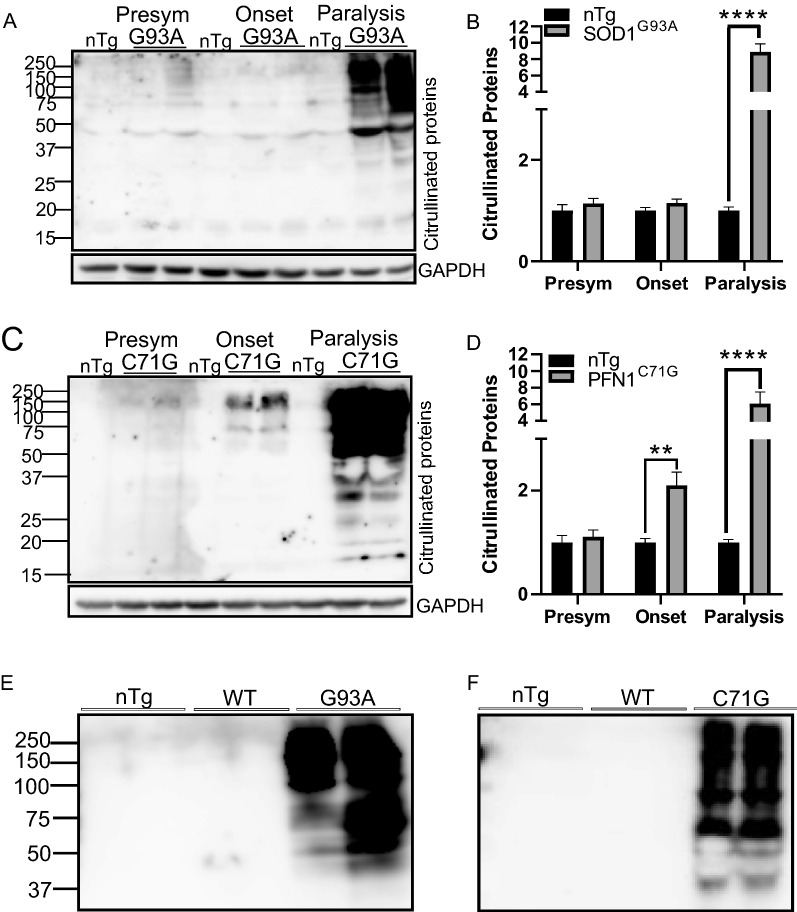
Protein citrullination (PC) is increased during disease progression. **A**, **C** Western blots of citrullinated proteins from lumber spinal cords of SOD1^G93A^ and PFN1^C71G^ ALS mice at different disease stages (see Methods for disease stage definition), and their age-matched non-transgenic (nTg) controls. **B**, **D** Quantification of lane signal density in blots as illustrated in **A** and **C**, respectively. n = 4 in all groups. Statistics: unpaired *t* test for comparing transgenic mice with their age matched controls. **p < 0.01; ****p < 0.0001. **E** Comparison of PC in the spinal cords of SOD1^G93A^ mice at paralysis stage with the age-matched nTg and SOD1 wild type (WT) transgenic mice. **F** Same as **E** but for PFN1^C71G^, PFN1^WT^ and nTg mice. Presym = presymptomatic

To determine whether PC is altered in ALS, we measured the levels of PC at different disease stages in two mouse models of ALS, SOD1^G93A^ and PFN1^C71G^. Citrullinated proteins were strongly increased in lumber spinal cord lysates during late disease stages (see Methods for disease stage definition) in both SOD1^G93A^ (Fig. 1A, B) and PFN1^C71G^ mouse models (Fig. 1C, D). These increases did not result from increased SOD1 or PFN1 activities, since citrullination did not change in the wild type SOD1 (Fig. 1E) and wild type PFN1 transgenic mice (Fig. 1F).

**Fig. 2:**
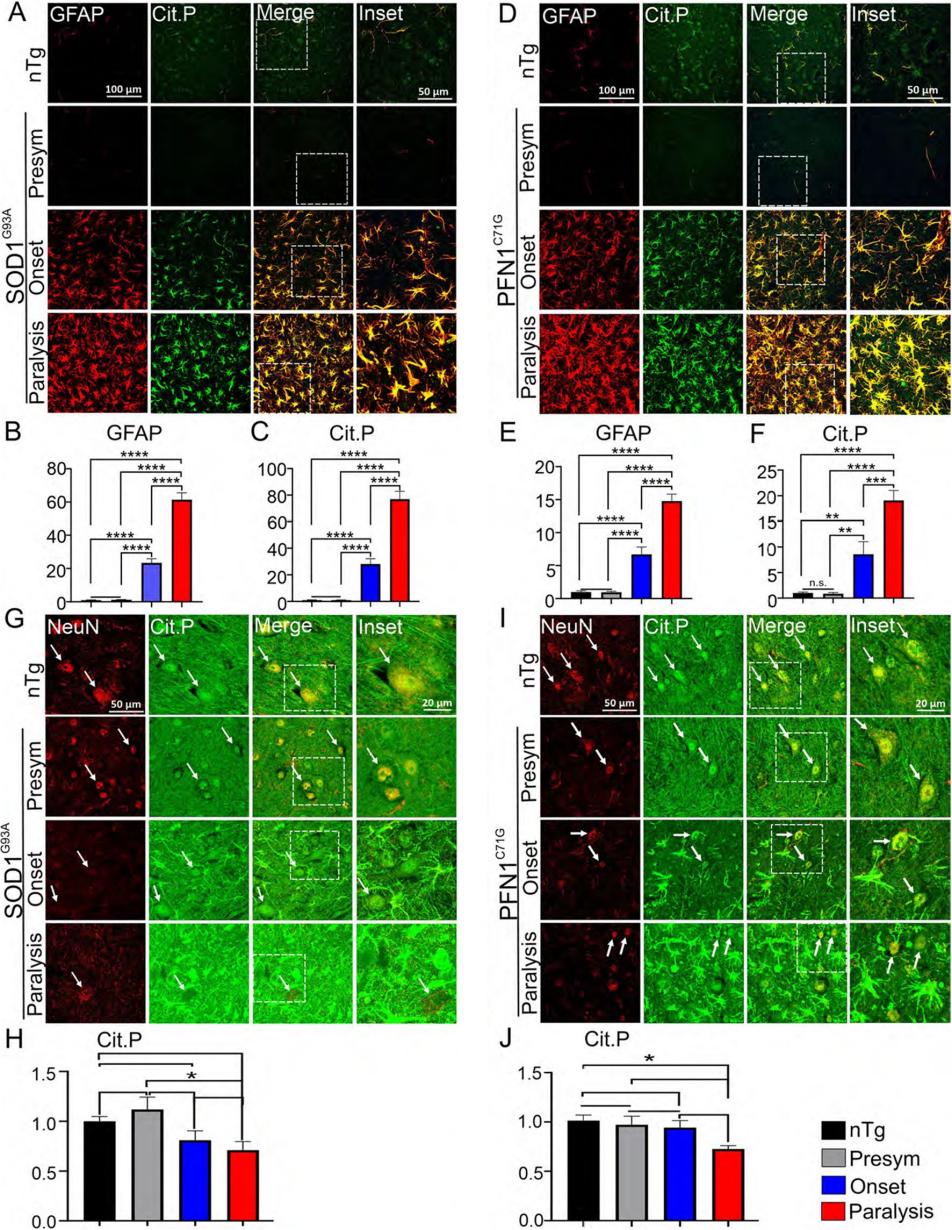
PC increases progressively in astrocytes but decreases in neurons in the spinal cord. **A** Double immunofluorescence staining for GFAP and citrullinated proteins in ventral horn in SOD1^G93A^ mice. **B**, **C** Quantification of fluorescent intensity of GFAP and citrullinated proteins, respectively, in **A**. **D** Double immunofluorescence staining for GFAP and citrullinated proteins in ventral horn in PFN1^C71G^ mice. **E**, **F** Quantification of fluorescent intensity of GFAP and citrullinated proteins, respectively, in **D**. **G** Double immunofluorescence staining for NeuN and citrullinated proteins in ventral horn in SOD1^G93A^ mice. **H** Quantification of fluorescent intensity of citrullinated proteins in NeuN-positive nuclei in **G**. **I** Double immunofluorescence staining for NeuN and citrullinated proteins in ventral horn in PFN1^C71G^ mice. **J** Quantification of fluorescent intensity of citrullinated proteins in NeuN-positive nuclei in **I**. The nTg mice used as controls for mutant SOD1 mice were ~ 150 days old. The nTg mice used as controls for mutant PFN1 mice were ~ 220 days old. For quantification, n = 8 in all groups. Statistics: one-way ANOVA with Bonferroni post-hoc test. *p < 0.05; **p < 0.01; ***p < 0.001; ****p < 0.0001. Unmarked pairs are not significantly different. Arrows point to examples of NeuN-positive and citrullinated protein-positive cells. Note the panels for citrullinated protein staining in **G**, **I** were overexposed in order to visualize the weak signals in neurons in paralysis stage of the disease

To determine the cell types where PC is increased, we performed double immunofluorescent staining for markers of various cell types and citrullinated proteins on the lumber spinal cord sections, followed by confocal microscopy. First, we investigated how PC is altered in astrocytes in the ventral horn by staining astrocytic marker GFAP. In the presymptomatic stage, staining signals for both GFAP and citrullinated proteins were relatively low (Fig. 2A, D), and not significantly different from the levels in the nTg mice (Fig. 2A–F). After disease onset, staining signals for GFAP and citrullinated proteins were increased progressively and dramatically (Fig. 2A–F). Over the course of the following weeks (~ 6 for SOD1^G93A^ mice and ~ 14 for PFN1^C71G^ mice), GFAP and citrullinated protein signals increased in parallel and were colocalized, spreading from intensely stained focal areas at disease onset to everywhere at the paralysis stage in the ventral horn (Fig. 2A–F, Additional file 2: Fig. S1).

Next, we investigated how PC was altered in neurons by staining neuronal marker NeuN. Because citrullinated protein staining was weak in neurons during disease progression, we used high exposure. In the presymptomatic stage, staining signal for citrullinated proteins was concentrated in the neuronal nucleus, like the NeuN signal (Fig. 2G, I). This pattern was the same as in the nTg mice (Fig. 2G, I). After disease onset, the citrullination signal displayed a decreasing trend in neurons, although statistical significance was only reached partially at the paralysis stage (Fig. 2H, J). To investigate PC in microglia and oligodendrocytes, we performed double immunofluorescent staining for citrullinated proteins with either IBA1 (microglia) or Olig2 (oligodendrocytes). The increased PC signal did not colocalize with either of these two markers (Additional file 2: Fig. S2, S3). Taken together, these results show that PC is increased in astrocytes but decreased in neurons during disease progression.

### PAD2 expression is altered in parallel with PC in the ventral horn

**Fig. 3:**
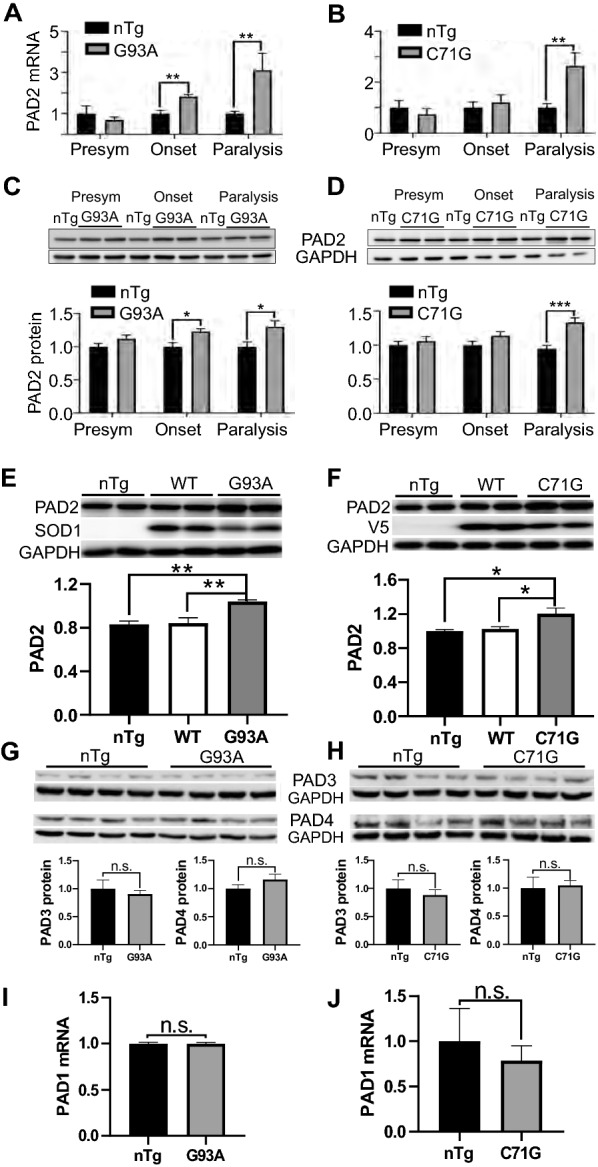
PAD2, but not PAD3, PAD4 and PAD1 expression, are increased. **A**, **B** Levels of PAD2 mRNA from lumber spinal cords of SOD1^G93A^ and PFN1^C71G^ mice at different disease stages and their age-matched nTg controls. **C**, **D** Western blots and quantification of PAD2 protein levels from lumber spinal cords of SOD1^G93A^ and PFN1^C71G^ mice at different disease stages and their age-matched nTg controls. **E** Comparison of PAD2 protein levels in the spinal cords of SOD1^G93A^ mice at paralysis stage with the age-matched nTg and SOD1 wild type (WT) transgenic mice. **F** Same as **E** but comparing PFN1^C71G^, PFN1^WT^, and nTg mice. Note that the endogenous SOD1 in the nTg mice was below detection at the loading amount. **G**, **H** Western blot and quantification for PAD3 and PAD4 in SOD1^G93A^ and PFN1^C71G^ mice, respectively. **I**, **J** PAD1 mRNA levels in SOD1^G93A^ and PFN1^C71G^ mice, respectively. For quantification, n = 4 in all groups. Statistics: unpaired *t* test. *p < 0.05; **p < 0.01. Unmarked pairs, or n.s., are not significantly different

Because PC is catalyzed by PADs, we next investigated whether PAD expression was altered. Because PAD2 is the dominant isoform in the CNS [15], we first examined its expression. Like PC, the levels of PAD2 mRNA were increased progressively in the spinal cords, by ~ threefold at the paralysis stage relative to the age-matched nTg controls (Fig. 3 A, B). PAD2 protein levels were elevated by ~ 30–40% in the mutant mouse models compared with the nTg controls (Fig. 3C, D). This increase was not related to increased SOD1 and PFN1 activities because PAD2 remained unchanged in wild type SOD1 and PFN1 transgenic mice (Fig. 3E, F). To determine whether the expression of other PAD isozymes is altered, we measured PAD3 and PAD4 protein levels and found no changes (Fig. 3G, H). Due to a lack of suitable antibodies, we measured PAD1 mRNA levels and did not find changes in the mutant mice (Fig. 3I, J). These results show that only PAD2 is upregulated during disease progression in ALS mouse models.

**Fig. 4:**
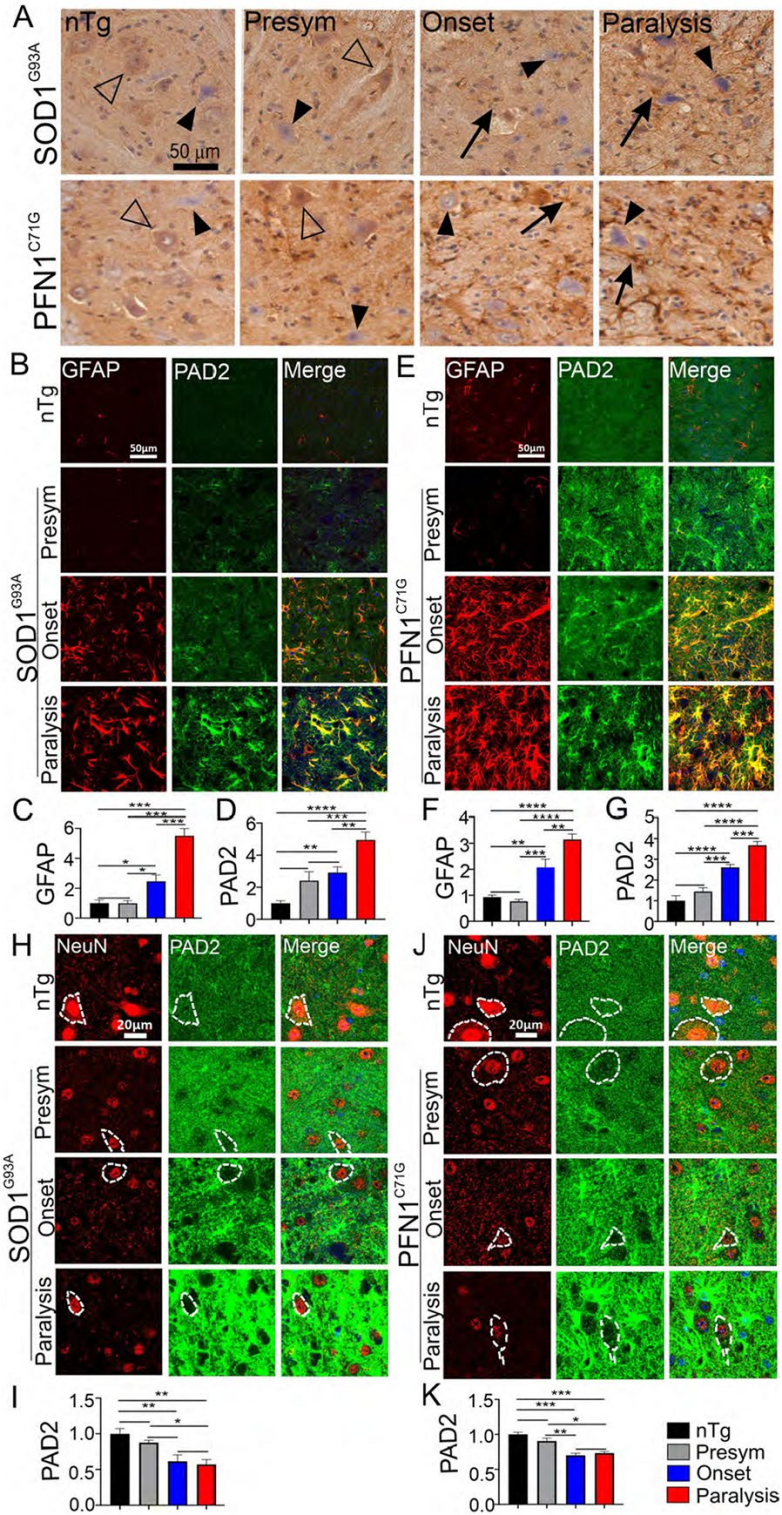
PAD2 expression is increased progressively in astrocytes but decreased in neurons in the spinal cord. **A** Immunohistochemistry staining of PAD2 in ventral horn spinal cords of SOD1^G93A^ and PFN1^C71G^ mice. Filled arrowheads point to PAD2-negative neurons, unfilled arrowheads point to PAD2-positive neurons, arrows point to PAD2-positive astrocytes. **B** Double immunofluorescence staining for GFAP and PAD2 in ventral horn in SOD1^G93A^ mice. **C**, **D** Quantification of fluorescent intensity of GFAP and PAD2, respectively, in **B**. **E** Double immunofluorescence staining for GFAP and PAD2 in ventral horn in PFN1^C71G^ mice. **F**, **G** Quantification of fluorescent intensity of GFAP and PAD2, respectively, in **E**. **H** Double immunofluorescence staining for NeuN and PAD2 in ventral horn in SOD1^G93A^ mice. **I** Quantification of fluorescent intensity of PAD2 staining in NeuN-positive cells in **H**. **J** Double immunofluorescence staining for NeuN and PAD2 in ventral horn in PFN1^C71G^ mice. **K** Quantification of fluorescent intensity of PAD2 staining in NeuN-positive cells in **J**. Note that the PAD2 staining was overexposed in **H**, **J** to ensure the visualization of weak signals in neurons. The ages of nTg mice are as described in Fig. 2. n = 8 in all groups. Statistics: one-way ANOVA with Bonferroni post-hoc test. *p < 0.05; **p < 0.01; ***p < 0.001; ****p < 0.0001. Comparison bars without (*) sign indicate not significant

To determine in which cell types PAD2 expression was upregulated, we surveyed the spinal cord ventral horn from mice at different disease stages by immunohistochemistry. During the presymptomatic stage, PAD2 expression was similar to the nTg controls. PAD2 was broadly expressed at various levels in motor neurons, with some slightly higher than the surrounding neuropils (Fig. 4A, open arrowheads) and others lower in both nTg and ALS mouse models (Fig. 4A, filled arrowheads). At disease onset, strong PAD2 staining emerged in astrocyte-like cells (Fig. 4A, arrows). At the paralysis stage, the strongly stained astrocytes became more numerous (see quantification below). The strong astrocytic staining contrasted with weakening neuronal staining, as the number of PAD2-negative neurons were increased (Fig. 4A, filled arrowheads).

To confirm and quantify these findings, we performed double immunofluorescent staining for PAD2 and GFAP, followed by confocal microscopy (Fig. 4B, E, Additional file 2: S4A, D). At presymptomatic stage, GFAP staining was not different from the nTg mice (Fig. 4C, F; Additional file 2: Fig. S4B, E); PAD2 staining was increased, although this increase did not reach statistical significance (Fig. 4D, G; Additional file 2: Fig. S4C, F). At disease onset and paralysis, both GFAP and PAD2 expression were increased significantly and were at the highest levels at the paralysis stage in the ALS models (Fig. 4B–G; Additional file 2: Fig. S4). The GFAP and PAD2 signals were mostly colocalized, thus indicating that PAD2 is upregulated predominantly in astrocytes. Because PC is also predominantly upregulated in astrocytes (Fig. 2), PAD2 and PC should be increased in the same cells. This was confirmed by double immunofluorescent staining for PAD2 and citrullinated proteins (Additional file 2: Fig. S5).

To investigate PAD2 expression in neurons, we performed double immunofluorescent staining for NeuN and PAD2. Like PC (Fig. 2G–J), PAD2 expression levels decreased as disease progressed in both ALS models (Fig. 4H–K). To investigate PAD2 expression in microglia and oligodendrocytes, we performed double immunofluorescent staining for PAD2 with either IBA1 (microglia) or Olig2 (oligodendrocytes). The increased PAD2 signal did not colocalize with either of these two markers (Additional file 2: Fig. S6, S7). Taken together, these results show that PAD2 is upregulated in spinal cord astrocytes and downregulated in neurons during disease progression in ALS mouse models, suggesting that the altered PAD2 expression is associated with changes in PC.

### Increased PAD2 expression and PC correlate with neurodegeneration

**Fig. 5:**
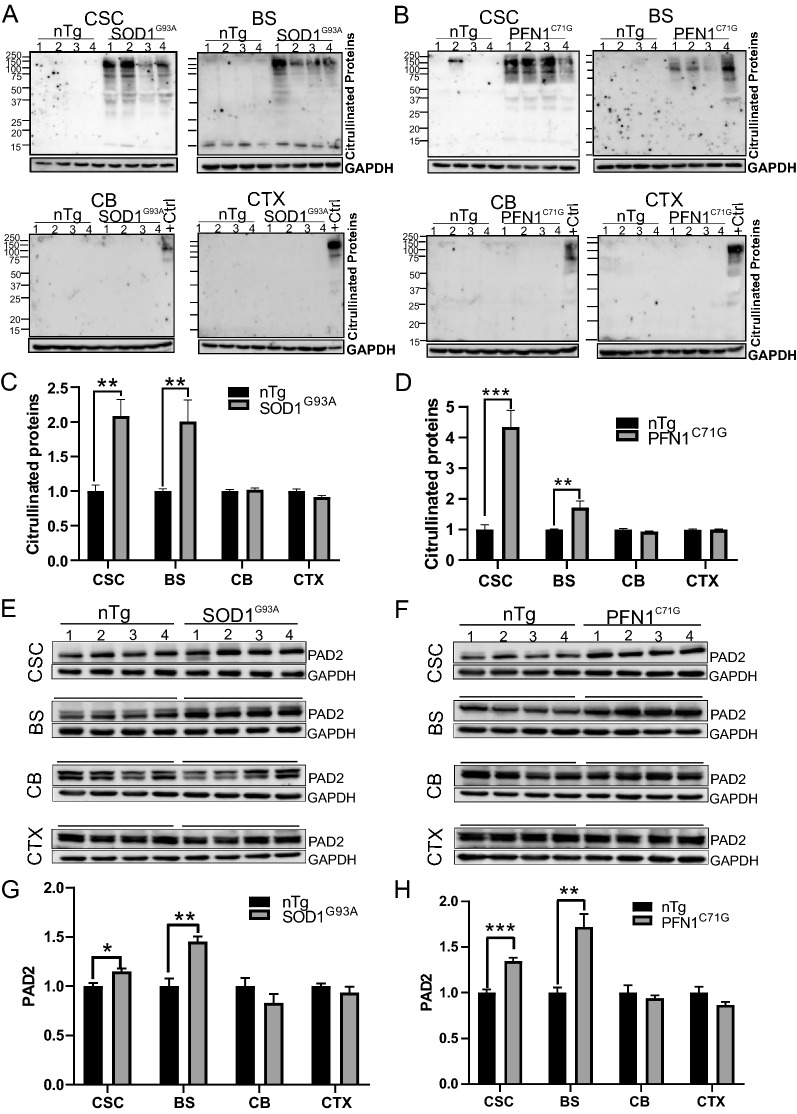
The increase of citrullinated proteins and PAD2 are correlated with regions of most motor neuron degeneration in the CNS. **A**, **B** Western blots of citrullinated proteins in cervical spinal cord (CSC), brainstem (BS), cerebellum (CB) and cortex (CTX) at paralysis stage in SOD1^G93A^ and PFN1^C71G^ mice, respectively. A CSC sample from paralysis stage was used as the positive control (+ Ctrl) in the cerebellum and cortex blots. **C**, **D** Quantification of citrullinated protein levels in **A**, **B** respectively. **E**, **F** Western blots of PAD2 protein in CSC, BS, CB, and CTX at paralysis stage in SOD1^G93A^ and PFN1^C71G^ mice, respectively. **G**, **H** Quantification of PAD2 protein levels in **E**, **F** respectively. n = 4 in each group. Statistics: unpaired *t* test for comparing transgenic mice with their age matched controls. *p < 0.05; **p < 0.01; ***p < 0.001. Unmarked pairs are not significantly different

The results described above show that PAD2, PC and GFAP increase in parallel during disease progression. Because GFAP is an astrogliosis marker and is correlated with neurodegeneration, we hypothesize that PAD2 and PC are also correlated with neurodegeneration. To test this, we measured levels of citrullinated proteins and PAD2 in various regions of the CNS, including cervical spinal cord (CSC), brainstem (BS), cerebellum (CB), and cerebral cortex (CTX). We found that in areas where severe neurodegeneration occurs, including lumbar spinal cord (LSC) (Fig. 1), CSC and BS (Fig. 5), PC and PAD2 were increased in both ALS models. In areas where there is minor or no neurodegeneration, such as CTX and CB, no increase in PC and PAD2 was detected (Fig. 5). These results indicate that the increased PC and PAD2 are associated with neurodegeneration.

### PC marks protein aggregates in spinal cord white matter

**Fig. 6:**
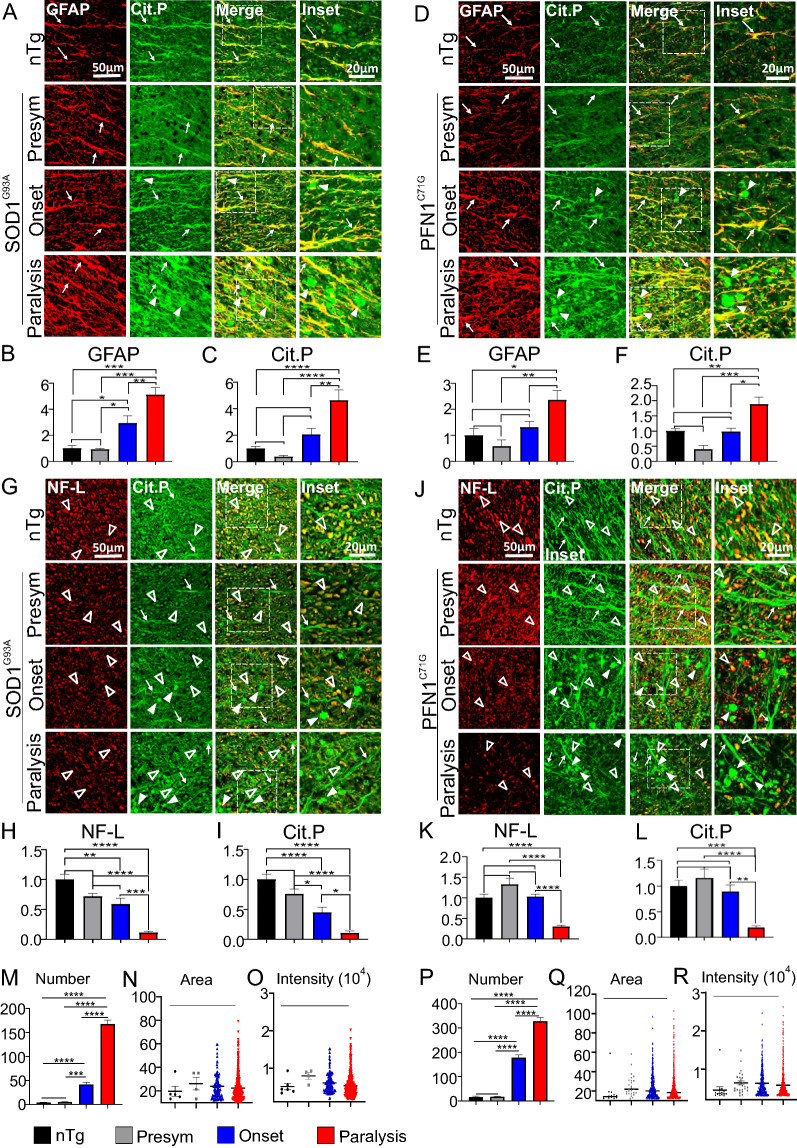
Citrullinated proteins accumulate in astrocytes and mark both axons and protein aggregates in the spinal cord white matter. **A**, **D** Double immunofluorescence staining for GFAP and citrullinated proteins in SOD1^G93A^ and PFN1^C71G^ mice, respectively. Arrows point to astrocytic processes that are positive for GFAP and citrullinated proteins. Arrowheads point to GFAP-negative but citrulline-positive aggregate-like structures. **B** Quantification of fluorescent intensity of GFAP staining in **A**. **C** Quantification of fluorescent intensity of citrullinated protein signal in areas that overlap with GFAP staining in **A**. **E** Quantification of fluorescent intensity of GFAP staining in **D**. Quantification of fluorescent intensity of citrullinated protein signal in areas that overlap with GFAP staining in **D**. **G**, **J** Double immunofluorescence staining for NF-L and citrullinated proteins in SOD1^G93A^ and PFN1^C71G^ mice, respectively. Arrows point to NF-L-negative and citrullinated protein-positive astrocytic processes. Filled arrowheads point to NF-L-negative but citrullinated-protein-positive aggregates. Unfilled arrowheads point to NF-L-positive and citrullinated protein-positive axons. **H** Quantification of fluorescent intensity of NF-L staining in **G**. **I** Quantification of fluorescent intensity of citrullinated protein signal in areas that overlap with NF-L staining in **G**. **K** Quantification of fluorescent intensity of NF-L staining in **J**. **L** Quantification of fluorescent intensity of citrullinated protein signal in areas that overlap with NF-L staining in **J**. **M**–**O** Number, area, and staining intensity of NF-L-negative and citrulline-positive aggregates, respectively, in SOD1^G93A^ mice. **P**–**R** Number, area, and staining intensity of NF-L-negative, citrulline-positive aggregates, respectively, in PFN1^C71G^ mice. The ages of nTg mice are as described in Fig. 2. For quantification in B, C, E, F, H-R, n = 6–7 in all groups. Statistics: One-way ANOVA with Bonferroni post-hoc test for comparison among the different groups. *p < 0.05; **p < 0.01; ***p < 0.001; ****p < 0.0001. Comparison bars lacking asterisks (*) indicate a lack of significant difference

Next, we examined PC in spinal cord white matter. Like the ventral horn, citrullinated proteins were colocalized with GFAP-positive astrocytic processes, and this GFAP-colocalized signal increased in parallel with GFAP as the disease progressed (Fig. 6A–F, arrows). However, we also noticed aggregate-like blobs that were strongly stained for citrullinated proteins and were not colocalized with GFAP (Fig. 6A, D, arrowheads). These blobs appeared to increase in number and size as the disease progressed to paralysis (for quantification, see below). Additionally, these structures did not contain PAD2 (Additional file 2: Fig. S5C, D, arrowheads).

To determine whether these aggregate-like blobs are associated with axons, we examined sections doubly stained for citrullinated proteins and neurofilament light-chain (NF-L). In the nTg mice, NF-L-positive axons appeared as dots of various sizes representing axons sectioned across, or short processes representing a short stretch of axons sectioned obliquely (Fig. 6G, J, open arrowheads). These dots and short processes were largely colocalized with citrullinated protein signal (Fig. 6G, J, open arrowheads), indicating that axonal proteins are citrullinated. We also observed citrulline-positive processes that were NF-L-negative (Fig. 6G, J, arrows). These are astrocytic processes as shown in Fig. 6A, D. In the presymptomatic stage of the ALS models, these structures were similar to the nTg (Fig. 6G, J). At the disease onset and paralysis stages, the NF-L signal became weaker, along with the citrullinated protein signals that were colocalized with the NF-L signal (Fig. 6G–L). Meanwhile, the citrullinated protein signal in citrulline-positive, but NF-L-negative, aggregate-like blobs appeared more numerous, larger, and more intense (Fig. 6G, J, filled arrowheads). A quantitative analysis confirms that these aggregate-like blobs grew in number, maximal size, and intensity, although the average size and intensity did not differ significantly among the nTg and ALS models at various disease stages (Fig. 6M–R).

**Fig. 7:**
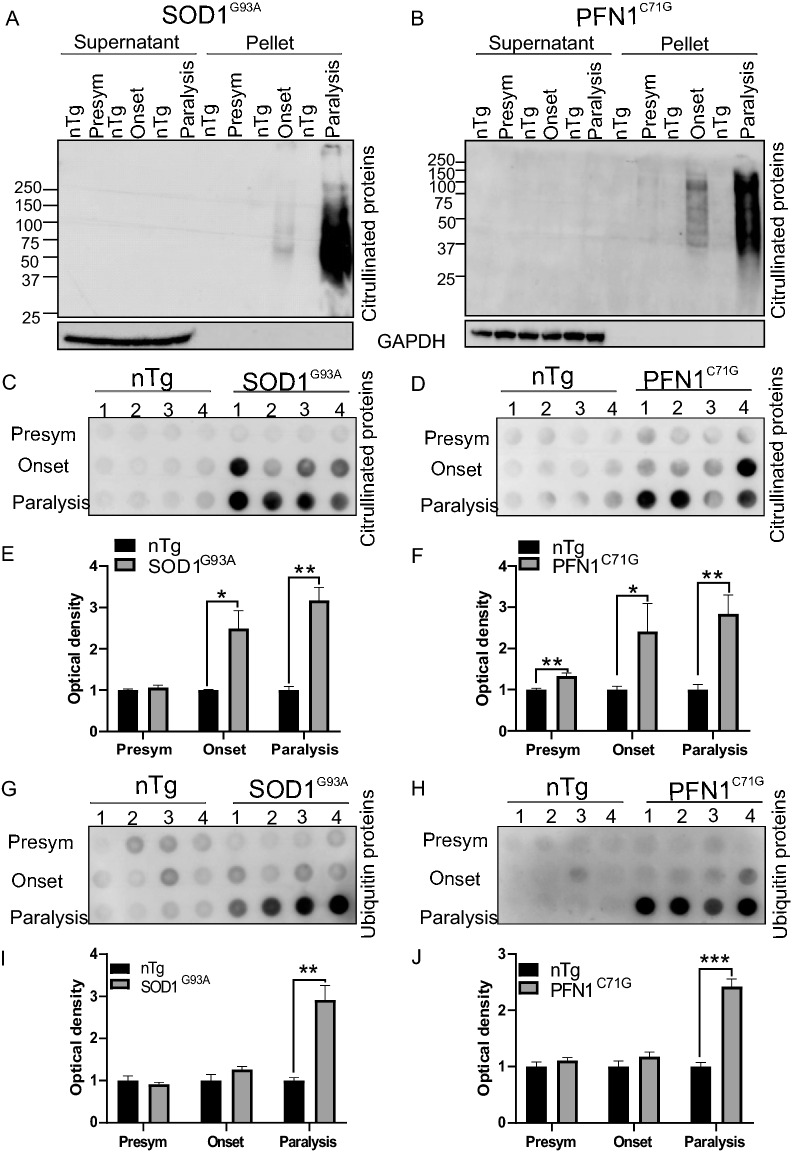
Citrullinated proteins are enriched in insoluble protein aggregates. **A**, **B** Sedimentation assay for citrullinated proteins in lumbar spinal cord. Homogenates from different disease stages and age-matched controls were centrifuged. Proteins in the supernatant and the pellet were resolved by SDS-PAGE. Citrullinated proteins were detected by the ACM method. **C**, **D** Filter trap assay for citrullinated protein aggregates from the spinal cord. **E**, **F** Quantification of optical density in **C**, **D**, respectively. **G**, **H** Filter trap assay for ubiquitinated protein aggregates. **I**, **J** Quantification of optical density in **G**, **H**, respectively. n = 4 in all groups. Statistics: unpaired *t* test for comparing transgenic mice with their age matched controls. *p < 0.05; **p < 0.01; ***p < 0.001

The above evidence suggests that citrullinated proteins are associated with protein aggregates. To confirm this, we performed a sedimentation assay (see methods). By this assay, citrullinated proteins were overwhelmingly enriched in the insoluble protein fraction, particularly in the late disease stage in the ALS models (Fig. 7A, B). To further confirm this finding, we conducted a filter trap assay (see methods) and detected abundant citrullinated proteins in protein aggregates in the ALS mouse models (Fig. 7C, D). Interestingly, significant elevations of citrullinated proteins were detected in the trapped protein aggregates in early disease stages of the ALS models (Fig. 7E, F).

The accumulation of ubiquitinated proteins in protein aggregates has been widely observed in neurodegenerative diseases [36]. To compare citrullinated and ubiquitinated proteins in the aggregates, we probed ubiquitinated proteins with the filter trap assay. As expected, ubiquitin also accumulated significantly, but only at the paralysis stage (Fig. 7G–J). This is later than the accumulation of the citrullinated proteins. Interestingly, the disease-triggering mutant proteins, SOD1^G93A^ and PFN1^C71G^, showed a similar, predominantly late-stage accumulation pattern, especially in PFN1^C71G^ ALS model (Additional file 2: Fig. S8). Taken together, these results suggest that PC is a PTM enriched in early disease stage protein aggregates, and its accumulation in protein aggregates precedes the accumulation of ubiquitination and the disease-causing mutant proteins.

### Citrullinated proteins show little colocalization with disease-associated mutant protein aggregates in spinal cord white matter

**Fig. 8:**
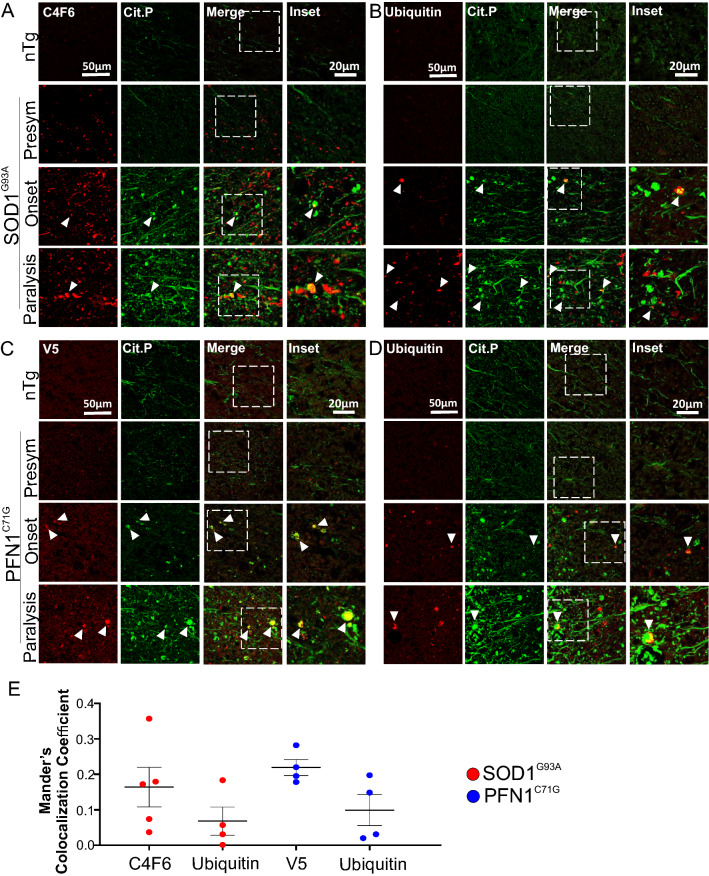
Most citrullinated protein aggregates do not colocalize with disease-specific mutant protein and ubiquitinated protein aggregates in the spinal cord white matter. Double immunofluorescence staining for **A** C4F6 and citrullinated protein in SOD1^G93A^ mice, **B** ubiquitin and citrullinated protein in SOD1^G93A^ mice, **C** V5 and citrullinated protein in PFN1^C71G^ mice, and **D** ubiquitin and citrullinated protein in PFN1^C71G^ mice. **E** Mander’s colocalization coefficient for citrullinated protein aggregates overlapping with disease-associated and ubiquitin-positive protein aggregates in SOD1^G93A^ and PFN1^C71G^ mice. The ages of nTg mice are as described in Fig. 2. Arrowheads point to C4F6-positive, V5-positive, or ubiquitin-positive citrullinated protein aggregates

Are citrullinated protein aggregates associated with the disease-causing, misfolded mutant protein aggregates? To answer this question, we doubly stained SOD1^G93A^ spinal cord sections for misfolded SOD1 protein using C4F6 antibody [37, 38], and citrullinated proteins. In the white matter, where most citrulline-positive aggregates reside, citrulline-positive aggregates showed little overlap with C4F6-positive aggregates (Manders colocalization coefficient: ~ 0.16; Fig. 8A, E, arrowheads). In the PFN1^C71G^ mice, we doubly stained spinal cord sections for V5 (tag for mutant PFN1) and citrullinated proteins. A similar low overlapping pattern was observed (Manders colocalization coefficient ~ 0.22; Fig. 8C, E, arrowheads).

To determine whether citrullinated protein aggregates colocalize with ubiquitin-positive aggregates, we doubly stained the spinal cord sections for ubiquitin and citrullinated proteins. Like the ALS-associated mutant protein aggregates, ubiquitin-positive aggregates showed little overlap with the citrulline-positive aggregates (Manders colocalization coefficient ~ 0.1; Fig. 8B, D, E). Overall, citrullinated protein aggregates are mostly independent from the aggregation of disease-associated mutant proteins and ubiquitin-modified proteins.

### Citrullinated proteins are highly colocalized with myelin protein PLP and MBP aggregates

**Fig. 9:**
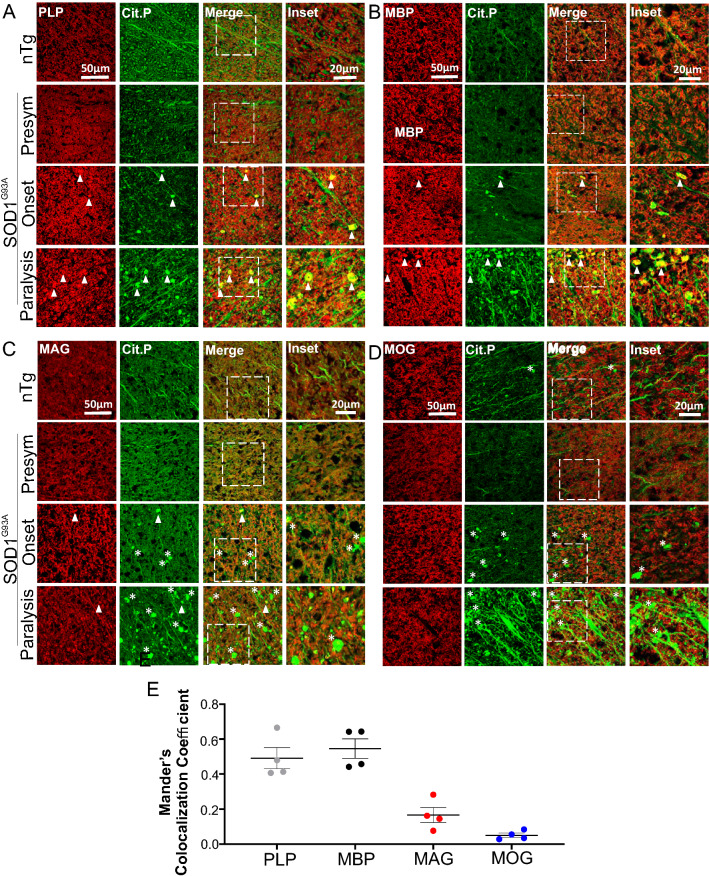
Citrullinated protein aggregates are highly colocalized with myelin proteins PLP and MBP and show less co-localization with MAG and MOG in the spinal cord white matter of SOD1^G93A^ mice. Double immunofluorescence staining for citrullinated proteins and PLP (**A**), MBP (**B**), MAG (**C**), and MOG (**D**). **E** Mander’s colocalization coefficient of citrullinated protein aggregates and PLP, MBP, MAG, and MOG, respectively. The ages of nTg mice are as described in Fig. 2. Arrowheads point to citrulline-positive protein aggregates that overlap with the myelin proteins. Asterisks mark citrulline-positive protein aggregates that do not overlap with the myelin proteins

**Fig. 10:**
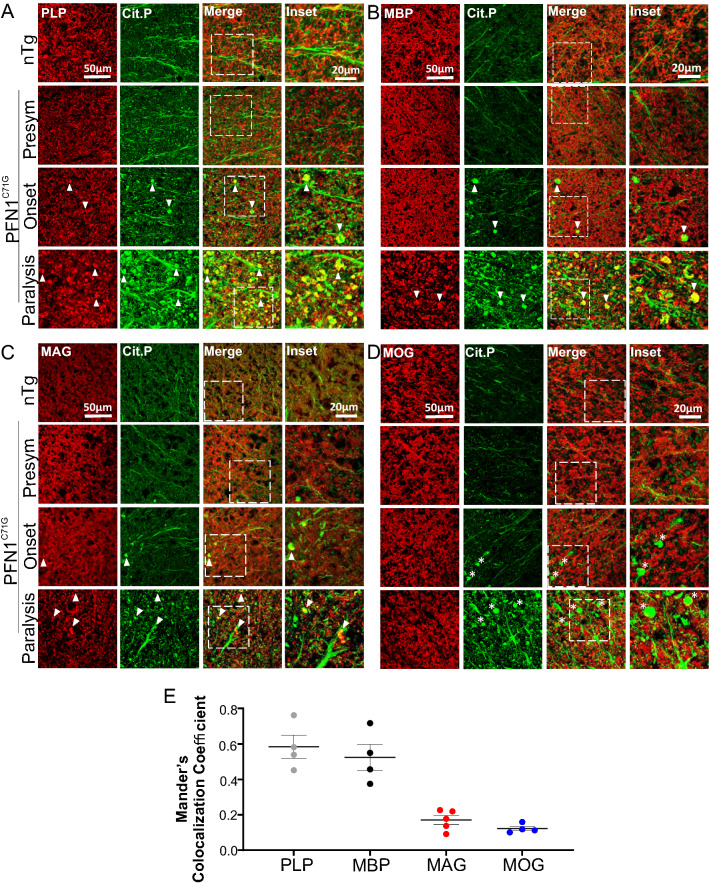
Citrullinated protein aggregates are highly colocalized with myelin proteins PLP and MBP, less with MAG and MOG in the spinal cord white matter of PFN1^C71G^ mice. Double immunofluorescence staining for citrullinated proteins and PLP (**A**), MBP (**B**), MAG (**C**), and MOG (**D**). **E** Mander’s colocalization coefficient of citrullinated protein aggregates and PLP, MBP, MAG, and MOG, respectively. The ages of nTg mice are as described in Fig. 2. Arrowheads point to citrulline-positive protein aggregates that overlap with the myelin proteins. Asterisks mark citrulline-positive protein aggregates that do not overlap with the myelin proteins

Because citrullinated protein aggregates are predominantly present in the white matter where myelinated axons are most abundant, we asked whether the citrullinated protein aggregates were associated with myelin. We performed double immunofluorescence analysis for PLP and citrullinated proteins on spinal cord sections. In nTg controls, PLP staining and citrullinated protein staining were separate. PLP showed a ring-like pattern, consistent with myelin structure (Figs. 9A, 10A). Citrullinated protein staining, on the other hand, showed a dot-like pattern, consistent with axons (Figs. 9A, 10A). The staining pattern in the presymptomatic stage was similar to nTg, indicating the myelinated axons were intact (Figs. 9A, 10A).

Upon disease onset, citrullinated protein aggregates began to emerge. These aggregates often contain PLP staining signal (Figs. 9A, 10A, arrowheads). As the disease progressed to the paralysis stage, citrullinated protein aggregates became more numerous, and these aggregates were highly colocalized with strong PLP staining signal (Figs. 9A, E, 10A, E). To confirm these findings, we doubly stained spinal cord sections for citrullinated proteins and various other myelin-associated proteins, including MBP, myelin associated glycoprotein (MAG), and myelin oligodendrocyte glycoprotein (MOG). MBP showed the same pattern as PLP (Figs. 9B, E, 10B, E). MAG staining revealed relatively few aggregates, which overlapped little with the citrulline-positive aggregates in both mouse models (Figs. 9C, E, 10C, E). MOG staining did not reveal convincing aggregates (Figs. 9D, E, 10D, E).

**Fig. 11:**
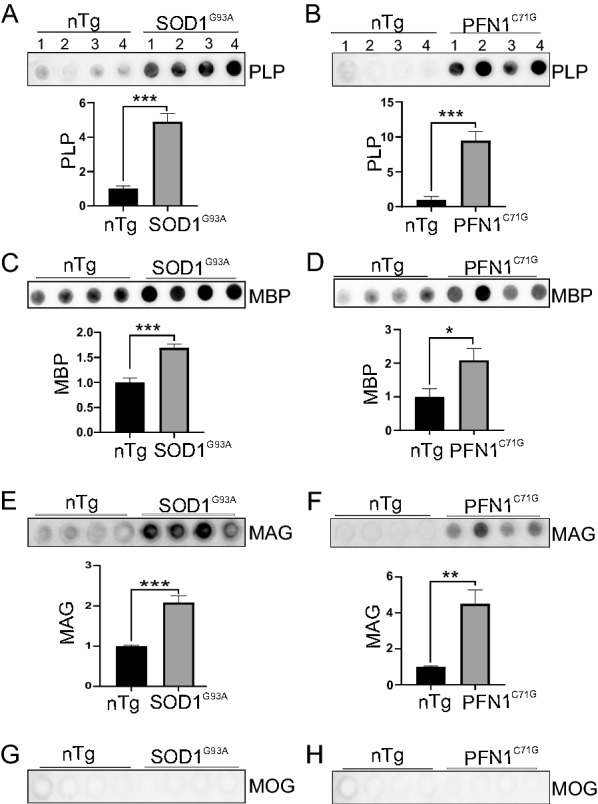
Myeline proteins, PLP, MBP, and MAG, but not MOG are enriched in insoluble protein aggregates in lumbar spinal cord. Filter trap assay and quantification of optical density of PLP (**A**, **B**), MBP (**C**, **D**), MAG (**E**, **F**), and MOG (**G**, **H**), in SOD1^G93A^ and PFN1^C71G^ ALS mice, respectively. All animals are at paralysis stage. n = 4 in each group. Statistics: unpaired *t* test for comparing transgenic mice with their age matched nTg controls. *p < 0.05; **p < 0.01; ***p < 0.001. n.s., not significant

To confirm the involvement of these myelin proteins in protein aggregation, we probed the myelin proteins using the filter trap assay. PLP, MBP, and MAG, but not MOG, accumulated significantly in both ALS models (Fig. 11A–H). Taken together, these results show PC are associated with PLP and MBP aggregates in ALS mouse models.

## Discussion

In this study, we investigated PC and PAD2 expression in two different ALS mouse models, one expressing human SOD1^G93A^ and the other PFN1^C71G^ mutant proteins. Our results show that PC is dynamically altered during disease progression in these mouse models. Whereas PC and PAD2 expression are generally increased in reactive astrocytes as reported in studies of other neurodegenerative disease models [20, 23, 39–41], our study has unveiled new, previously unknown changes. We demonstrate that, in contrast to the increase in astrocytes, PC and PAD2 expression trend downwards in neurons as disease progresses (Figs. 2, 4). Additionally, we show that the overall increases in PC and PAD2 expression correlate temporally with disease progression and spatially with the areas of severe neurodegeneration (Figs. 1, 5). Most strikingly, we show that citrullinated proteins are overwhelmingly enriched in protein aggregates and predominantly colocalized with myelin protein PLP and MBP (Figs. 6, 7, 8, 9, 10), suggesting that citrullinated proteins are a new class of proteins that aggregate in the white matter in ALS.

Abnormal PAD activation and accumulation of PC have been documented in several neurodegenerative diseases including AD, PD, MS, and prion diseases [18, 24, 26, 42]. Our results suggest that ALS also belongs to this list, although confirmation with human samples will be needed. By longitudinal observation along disease progression, we show that, temporally, increased levels of PC and PAD2 emerged at disease onset, and then accumulated progressively, culminating at the paralysis stage (Figs. 1, 3). By examination of different CNS areas, we show that, spatially, both PC and PAD2 accumulated in areas of severe neurodegeneration (Fig. 5). These patterns parallel the changes in other hallmarks of ALS pathology, including motor neuron loss, reactive gliosis, and accumulation of protein aggregates in these mouse models [32, 43, 44]. The citrullination changes also draw similarities with other well-established PTM protein neuropathological markers, such as phosphorylated tau in AD and frontotemporal dementia (FTD), and phosphorylated TDP-43 in ALS and FTD. The appearance and subsequent accumulation of these markers have been shown to correlate temporally with disease progression and spatially with the disease-specific spread pattern in the CNS [45–47]. Thus, our results from both ALS models suggest that citrullinated proteins could be another robust PTM protein marker for ALS and possibly other neurodegenerative conditions.

Previous studies have shown that the increases in PC and PAD2 are most prominently displayed in astrocytes during neurodegeneration [18, 19, 39, 48, 49]. Our results have unveiled new details in the ALS mouse models. PC and PAD2 accumulate in parallel with GFAP in astrocytes in the ALS mouse models, initially focally and later widespread (Additional file 2: Fig. S1). By contrast, PC and PAD2 expression are decreased in neurons (Figs. 2, 4), suggesting that PC changes are not uniform and occur in a cell type-specific manner. How these changes contribute to neurodegeneration is not yet known. However, PC alters protein functions [9, 14, 50]. Furthermore, polypeptides relevant to neurodegeneration, such as amyloid β peptide, GFAP, vimentin, FET proteins, myelin proteins, and others, have been shown to be citrullinated [9, 14, 51, 52]. Therefore, we speculate that PC in the ALS mouse models alters protein function and modulate the disease. The divergent changes in astrocytes vs. neurons suggest that the functional effects of PC alterations would likely be multifaceted and their effects on the disease complex. For example, some citrullination changes may antagonize disease progression, while other changes promote the disease. Future studies that identify the citrullinated proteins and the sites, and functional tests of the citrullination will be required to determine the effects on the proteins and on the disease.

Protein aggregation is associated with neurodegenerative diseases, although the role of protein aggregation in neurodegeneration remains uncertain [53, 54]. Some post-translationally modified proteins are highly enriched in protein aggregates in neurodegenerative diseases, e.g., phosphorylated tau in AD and FTD, and phosphorylated TDP-43 in ALS. These proteins have been used as specific markers for protein aggregates in these diseases. Quantitative measurements of the spatial and temporal spread of the protein aggregates labeled with these markers have been adopted to classify the disease stages, helping to unveil the pathological evolution of the disease in the CNS [45–47]. In this study, we found that citrullinated proteins are concentrated in intensely stained blobs in the spinal cord white matter (Figs. 6, Additional file 2: S5). Subsequently, we showed that citrullinated proteins are highly enriched in the insoluble proteins by sedimentation and filter trap assays (Fig. 7), thus confirming that citrullinated proteins are in the protein aggregates. These results indicate that citrullinated proteins are a new marker for protein aggregates in the white matter in the ALS mouse models. Further studies will be required to determine whether citrullinated proteins can be used as a marker for protein aggregates and possibly as a marker for classifying white matter degeneration in human ALS.

Can citrullination drive protein aggregation? The answer is unknown. Studies on other PTMs, such as phosphorylated tau in AD and FTD, and phosphorylated TDP-43 in ALS, have shown nuanced or contradictory results. In tau, there are numerous phosphorylated sites. Whereas phosphorylation at some sites promote aggregation, phosphorylation at other sites appear to inhibit aggregation [55, 56]. In TDP-43, phosphorylation at the c-terminal S409 and S410 are highly enriched in protein aggregates [57–59]. However, whether phosphorylation drives aggregation remains controversial [57, 60–62]. For PC, heavy artificial citrullination in vitro can denature proteins, which can lead to protein aggregation [63]. Another example may be MBP. Previous studies have shown that citrullination causes its dissociation from membrane (see below). Because MBP is an intrinsically disordered protein and has an extended and unfolded structure when it is not bound to membrane [64], the dissociation from the membrane could lead to its aggregation. Further studies will be needed to determine whether citrullination drives protein aggregation.

An interesting finding in this study is that the white matter protein aggregates scarcely overlapped with the aggregates formed by the disease-causing mutant proteins, SOD1 and PFN1 (Fig. 8). Instead, the white matter protein aggregates are heavily citrullinated and contain abundant myelin proteins, PLP and MBP (Figs. 9, 10, 11). PLP and MBP are two of the most abundant proteins in myelin and are estimated to constitute ~ 70% of total proteins in myelin [65]. Their involvement in the aggregates suggests that these proteins may be the core of the white matter protein aggregates. The fact that PLP and MBP form aggregates independently from the mutant PFN1 or SOD1 suggest that these protein aggregations are driven by different pathways from the mutant protein aggregation and are not induced by copying the misfolded and aggregated conformation of the mutants. Previous studies have shown that aggregates formed by multiple proteins can coexist, either separately or co-aggregate together, in individual cases of neurodegenerative disease and in animal models [54]. In AD, Aβ plaques coexist with tau tangles, α-synuclein or TDP-43 aggregates; in PD and other synucleinopathies, α-synuclein aggregates coexist with aggregates composed of Aβ, tau or TDP-43; and in ALS, TDP-43 aggregates can coexist with other aggregates formed by various proteins, including VCP, FUS, ATXN2, RBM45, P62, OPTN, TIA1, and others [66–68]. However, whether these aggregates are formed by cross-seeding each other or by other mechanisms remains unclear [54].

What pathway underlies formation of myelin protein aggregates? Based on our observations, we postulate that citrullination plays a central role. Although the citrullination status of PLP remains unclear, citrullination of MBP is well established. MBP is citrullinated in normal myelin and, in pathological conditions such as MS, MBP citrullination is dramatically increased. MBP carries strong positive charges partly due to its abundant arginines. These positive charges enable MBP to bind negatively charged phospholipids in the membrane and compact myelin membrane layers. The increased citrullination in MS reduces the positive charges in MBP, thereby reducing binding to the negatively charged membrane. The membrane-unbound MBP has an open and unfolded structure and subsequent proteolysis releases immunodominant peptides, triggering the autoimmune response and destruction of myelin [11, 69, 70]. A variation of this mechanism could be at play in ALS. Citrullination of MBP (and possibly PLP) could be increased in ALS, resulting in its dissociation from the myelin membrane, misfolding, and aggregation. This process could contribute to myelin degeneration in ALS. Further experiments will be required to test the validity of this hypothesis.

In summary, this study demonstrates that PC is altered dynamically during disease progression in two mouse models for ALS. While citrullination is increased in astrocytes, it is decreased in neurons as disease symptoms progress. The large increase in astrocytes dominates the detected citrullination changes and this increase correlates temporally and spatially with disease progression, a characteristic that may be adopted as a new pathological marker for the disease progression. Furthermore, we show that citrullinated proteins are enriched in protein aggregates that are predominantly located in white matter and colocalized with myelin proteins PLP and MBP, suggesting that citrullination of myelin proteins may lead to myelin protein aggregation and contribute to myelin degeneration in ALS. These results indicate that further studies are needed to dissect the roles that PC play in the pathogenesis of ALS.

## Supplementary Information

**Additional file 1: Table S1.** List of Primary Antibodies used in the Study. **Table S2.** List of Secondary Antibodies used in the Study.

**Additional file 2: Figure S1.** Citrullinated proteins accumulate as foci in reactive astroglia (dotted circles) in early disease stages and become widespread in late disease stages in ALS mouse models. (A, B) Double immunofluorescence staining for GFAP and citrullinated protein in ventral horn spinal cords of SOD1^G93A^ and PFN1^C71G^ mice, respectively. **Figure S2**. PC is not increased in microglia in the spinal cord of ALS mouse models. (A, B) Double immunofluorescence staining for IBA1 and citrullinated proteins in the ventral horn gray matter of SOD1^G93A^ and PFN1^C71G^ mice, respectively. (C, D) Double immunofluorescence staining for IBA1 and citrullinated proteins in the ventral lateral white matter of SOD1^G93A^ and PFN1^C71G^ mice, respectively. The ages of nTg mice are as described in Fig. 2. **Figure S3.** PCs are not increased in oligodendrocytes in the spinal cord of ALS mouse models. (A, B) Double immunofluorescence staining for Olig2 and citrullinated proteins in ventral horn gray matter of SOD1^G93A^ and PFN1^C71G^ mice, respectively. (C, D) Double immunofluorescence staining for Olig2 and citrullinated proteins in the ventral lateral white matter of SOD1^G93A^ and PFN1^C71G^ mice, respectively. The ages of nTg mice are as described in Fig. 2. **Figure S4.** PAD2 expression is increased progressively in astrocytes in the spinal cord white matter of ALS mouse models. (A) Double immunofluorescence staining for GFAP and PAD2 in spinal cord white matter in SOD1^G93A^ mice. (B, C) Quantification of fluorescent intensity of GFAP and PAD2, respectively, in (A). (D) Double immunofluorescence staining for GFAP and PAD2 in spinal cord white matter in PFN1^C71G^ mice. (E, F) Quantification of fluorescent intensity of GFAP and PAD2, respectively, in (D). The ages of nTg mice, n, and statistics are as described in Fig. 2. **Figure S5.** PAD2 and citrullinated proteins are colocalized in astrocytes but not in aggregates in the spinal cord of ALS mouse models. (A, B) Double immunofluorescence staining for PAD2 and citrullinated proteins in the ventral horn gray matter of SOD1^G93A^ and PFN1^C71G^ mice, respectively. (C, D) Double immunofluorescence staining for PAD2 and citrullinated proteins in the ventral lateral white matter of SOD1^G93A^ and PFN1^C71G^ mice, respectively. Arrowheads point to PAD2-negative but citrulline-positive protein aggregates. The ages of nTg mice are as described in Fig. 2. **Figure S6**. PAD2 expression is not increased in microglia in the spinal cord of ALS mouse models. (A, B) Double immunofluorescence staining for IBA1 and PAD2 in ventral horn gray matter of SOD1^G93A^ and PFN1^C71G^ mice, respectively. (C, D) Double immunofluorescence staining for IBA1 and PAD2 in ventral lateral white matter of SOD1^G93A^ and PFN1^C71G^ mice, respectively. The ages of nTg mice are as described in Fig. 2. The blue color in the merge panel represents the nucleus as stained by DAPI. **Figure S7.** PAD2 expression is not increased in oligodendrocytes in the spinal cord of ALS mouse models. (A, B) Double immunofluorescence staining for Olig2 and PAD2 in ventral horn gray matter of SOD1^G93A^ and PFN1^C71G^ mice, respectively. (C, D) Double immunofluorescence staining for Olig2 and PAD2 in ventral lateral white matter of SOD1^G93A^ and PFN1^C71G^ mice, respectively. The ages of nTg mice are as described in Fig. 2. The blue color in the merge panel represents the nucleus as stained by DAPI. **Figure S8.** Disease-specific protein aggregates are observed in spinal cords from ALS mouse models. (A) Filter trap assay and quantification of mutant SOD1 protein aggregation. (B) Filter trap assay and quantification of mutant PFN1 protein aggregation. n = 4 in each group. Statistics: unpaired t-test for comparing transgenic mice with their age matched controls. *p < 0.05. ****p < 0.0001. **Figure S9.** Microscopy and Image processing workflow for quantitative analyses of immunofluorescence-stained spinal cord sections. (A) An example for quantifying PAD2 fluorescent intensity in astrocytes in ventral horn gray matter of a paralyzed PFN1^C71G^ mouse. GFAP was used as a marker to identify astrocytes. The GFAP (red) image was thresholded using ImageJ, and GFAP-positive areas were identified as regions of interest (ROI). The ROI was overlayed on the corresponding immunostained PAD2 (green) image, and PAD2 fluorescent intensity was quantified exclusively within the defined ROI. (B) As in (A) but showing white matter. (C) An example of quantifying citrullinated protein fluorescent intensity in axons of nTg spinal cord white matter. NF-L was used as a marker to identify axons. The NF-L (Green) image was thresholded and NF-L-positive areas were identified as ROI. These ROIs were overlayed on the immunostained citrullinated proteins (red) image, and citrullinated protein fluorescent intensity was quantified exclusively within the defined ROI. (D) Same as (C) but in white matter of a paralyzed PFN1^C71G^ mouse. **Figure S10.** Procedure for colocalization analysis in the spinal cord of ALS mouse models. Levels of colocalization between citrullinated protein aggregates and other proteins were analyzed using JACoP plugin in ImageJ. (A) PLP- and citrullinated protein-stained images (top) were thresholded to remove background and other weakly stained signals (bottom). (B) The Costes’ mask was generated showing colocalization (white), background (black), PLP (red), and citrullinated proteins (green). The result (colocalization) was expressed as Manders’ overlap coefficient (M2).

## Data Availability

All data supporting the conclusions of this article are included within the article and its supplementary materials.
