# Supplementary material for: Protein citrullination marks myelin protein aggregation and disease progression in mouse ALS models
Source: Acta Neuropathol Commun. 2022 Sep 8;10:135. doi: 10.1186/s40478-022-01433-5 (PMC9458309; doi:10.1186/s40478-022-01433-5)
Supplement: Supplementary file 1 — Additional file 1: Table S1. List of Primary Antibodies used in the Study. Table S2. List of Secondary Antibodies used in the Study. [file 40478_2022_1433_MOESM1_ESM.pdf]

Supplementary Table 1:

List of Primary Antibodies used in the Study.

**Antibody**	**Company**	**Catalog No.**	**Dilution**	**RRID**
PADI2	Proteintech	12110-1-AP	1:1000 (WB)<br>1:50 (IF)	AB_2159475
Anti-Modified citrulline, clone C4 (human recombinant)	Millipore	MABS487	1:1000 (WB)<br>1:50 (IF)	
PADI4	Proteintech	17373-1-AP	1:1000 (WB)	AB_2878398
PADI3 (F-6)	Santacruz	sc-393622	1:100 (WB)	
GFAP	Cell Signalling	3670S	1:400 (IF)	AB_561049
GAPDH (6C5)	Santacruz	Sc-32233	1:1000 (WB)	AB_627679
Anti-NeuN	Millipore	MAB377	1:50 (IF)	AB_2298772
AIF-1/Iba1	Novus Biologicals	NB100-1028	1:100 (IF)	AB_521594
Anti-NF-L	Zuoshang		1:300 (IF)	
Myelin PLP	Novus Biologicals	NB100-1608	1:100 (IF)	AB_2062362
Anti-MBP, a.a. 82-87	Millipore	MAB386	1:200 (IF)	AB_94975
MAG (A-11)	Santacruz	sc-166849	1:100 (IF)	AB_2250078
MOG (D-2)	Santacruz	sc-376138	1:100 (IF)	AB_10989782
V5 Tag	Bethyl Laboratories	A190-119A	1:400 (IF)<br>1:1000 (WB)	AB_67317
Anti-SOD1	Non-commercial	BioDesign, Saco, ME, USA	1:1000 (WB)	
Misfolded SOD1 (C4F6)	From Dr. Daryl Bosco, University of Massachusetts Medical School	Non-commercial	1:200 (IF)	
Profilin (Ser-138)	ECM BIOSCIENCES	PP4791	1:250 (IF)	
Ubiquitin	Dako	Z0458	1:400 (IF)<br>1:1000 (WB)	AB_2315524
CNPase (H-2)	Santacruz	Sc-166558	1:100 (IF)	AB_2082602

Supplementary Table 2:

List of Secondary Antibodies used in the Study.

Antibody	Catalog No.	Company	Dilution	RRID
Goat anti-Rabbit IgG (H+L), (HRP)	65-6120	Invitrogen	1:5000 (WB)	AB_2533967
Goat anti-Mouse IgG (H+L), (HRP)	62-6520	Thermo Fisher Scientific	1:5000 (WB)	AB_2533947
Rabbit anti-Sheep IgG, H & L Chain Specific HRP Conjugate	402100	Millipore	1:5000 (WB)	AB_437820
Goat Anti-Human IgG, HRP Conjugate	CS216591	Millipore	1:2000 (WB)	
Donkey anti-Goat IgG (H+L), (HRP)	A15999	Invitrogen	1:5000 (WB)	AB_2534673
Alexa Fluor® 488-conjugated AffiniPure Goat Anti-Human IgG (H+L)	109-545-003	Jackson Immuno Research Laboratories	1:250 (IF)	AB_2337831
AffiniPure Goat Anti-Human IgG (H+L). Dylight™ 549	109-505-088	Jackson Immuno Research Laboratories	1:250 (IF)	AB_2337539
Alexa Fluor™ 488 donkey anti-mouse IgG (H+L)	A21202	Thermo Fisher Scientific	1:250 (IF)	AB_141607
Alexa Fluor® 568 donkey anti-mouse IgG (H+L)	A10037	Thermo Fisher Scientific	1:250 (IF)	AB_2534013
Alexa Fluor™ 568 donkey anti-rabbit IgG (H+L)	A10042	Thermo Fisher Scientific	1:250 (IF)	AB_2534017
Alexa Fluor® 568 goat anti-mouse IgG (H+L)	A11031	Thermo Fisher Scientific	1:250 (IF)	AB_144696
Alexa Fluor™ 488 donkey anti-rabbit IgG (H+L)	A21206	Thermo Fisher Scientific	1:250 (IF)	AB_2535792
Alexa Fluor® 488 goat anti-rabbit IgG (H+L)	A11034	Thermo Fisher Scientific	1:250 (IF)	AB_2576217
Alexa Fluor® 594 goat anti-rabbit IgG (H+L)	A11037	Thermo Fisher Scientific	1:250 (IF)	AB_2534095
Alexa Fluor® 488 goat anti-mouse IgG (H+L)	A11029	Thermo Fisher Scientific	1:250 (IF)	AB_2534088
Alexa Fluor® 568 donkey anti-goat IgG (H+L)	A11057	Thermo Fisher Scientific	1:250 (IF)	AB_2534104
