# Supplementary material for: Protein citrullination marks myelin protein aggregation and disease progression in mouse ALS models
Source: Acta Neuropathol Commun. 2022 Sep 8;10:135. doi: 10.1186/s40478-022-01433-5 (PMC9458309; doi:10.1186/s40478-022-01433-5)
Supplement: Supplementary file 2 — Additional file 2: Figure S1. Citrullinated proteins accumulate as foci in reactive astroglia (dotted circles) in early disease stages and become widespread in late disease stages in ALS mouse models. (A, B) Double immunofluorescence staining for GFAP and citrullinated protein in ventral horn spinal cords of SOD1G93A and PFN1C71G mice, respectively. Figure S2. PC is not increased in microglia in the spinal cord of ALS mouse models. (A, B) Double immunofluorescence staining for IBA1 and citrullinated proteins in the ventral horn gray matter of SOD1G93A and PFN1C71G mice, respectively. (C, D) Double immunofluorescence staining for IBA1 and citrullinated proteins in the ventral lateral white matter of SOD1G93A and PFN1C71G mice, respectively. The ages of nTg mice are as described in Fig. 2. Figure S3. PCs are not increased in oligodendrocytes in the spinal cord of ALS mouse models. (A, B) Double immunofluorescence staining for Olig2 and citrullinated proteins in ventral horn gray matter of SOD1G93A and PFN1C71G mice, respectively. (C, D) Double immunofluorescence staining for Olig2 and citrullinated proteins in the ventral lateral white matter of SOD1G93A and PFN1C71G mice, respectively. The ages of nTg mice are as described in Fig. 2. Figure S4. PAD2 expression is increased progressively in astrocytes in the spinal cord white matter of ALS mouse models. (A) Double immunofluorescence staining for GFAP and PAD2 in spinal cord white matter in SOD1G93A mice. (B, C) Quantification of fluorescent intensity of GFAP and PAD2, respectively, in (A). (D) Double immunofluorescence staining for GFAP and PAD2 in spinal cord white matter in PFN1C71G mice. (E, F) Quantification of fluorescent intensity of GFAP and PAD2, respectively, in (D). The ages of nTg mice, n, and statistics are as described in Fig. 2. Figure S5. PAD2 and citrullinated proteins are colocalized in astrocytes but not in aggregates in the spinal cord of ALS mouse models. (A, B) Double immunofluorescence staining f [file 40478_2022_1433_MOESM2_ESM.pdf]

Figure S1

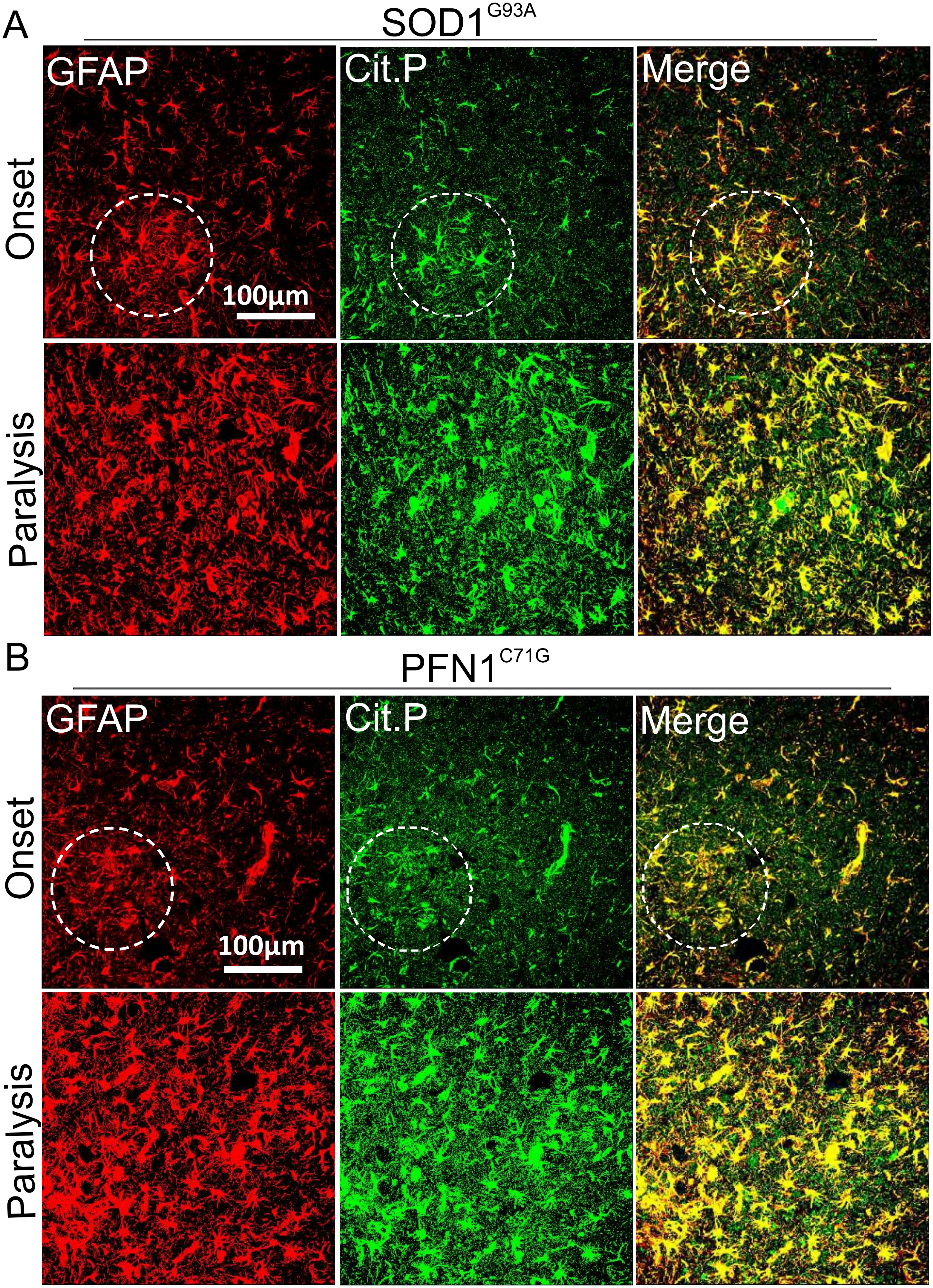
A
SOD1^{G93A}
GFAP
Cit.P
Merge
Onset
Paralysis
100µm
B
PFN1^{C71G}
GFAP
Cit.P
Merge
Onset
Paralysis
100µm

Figure S2

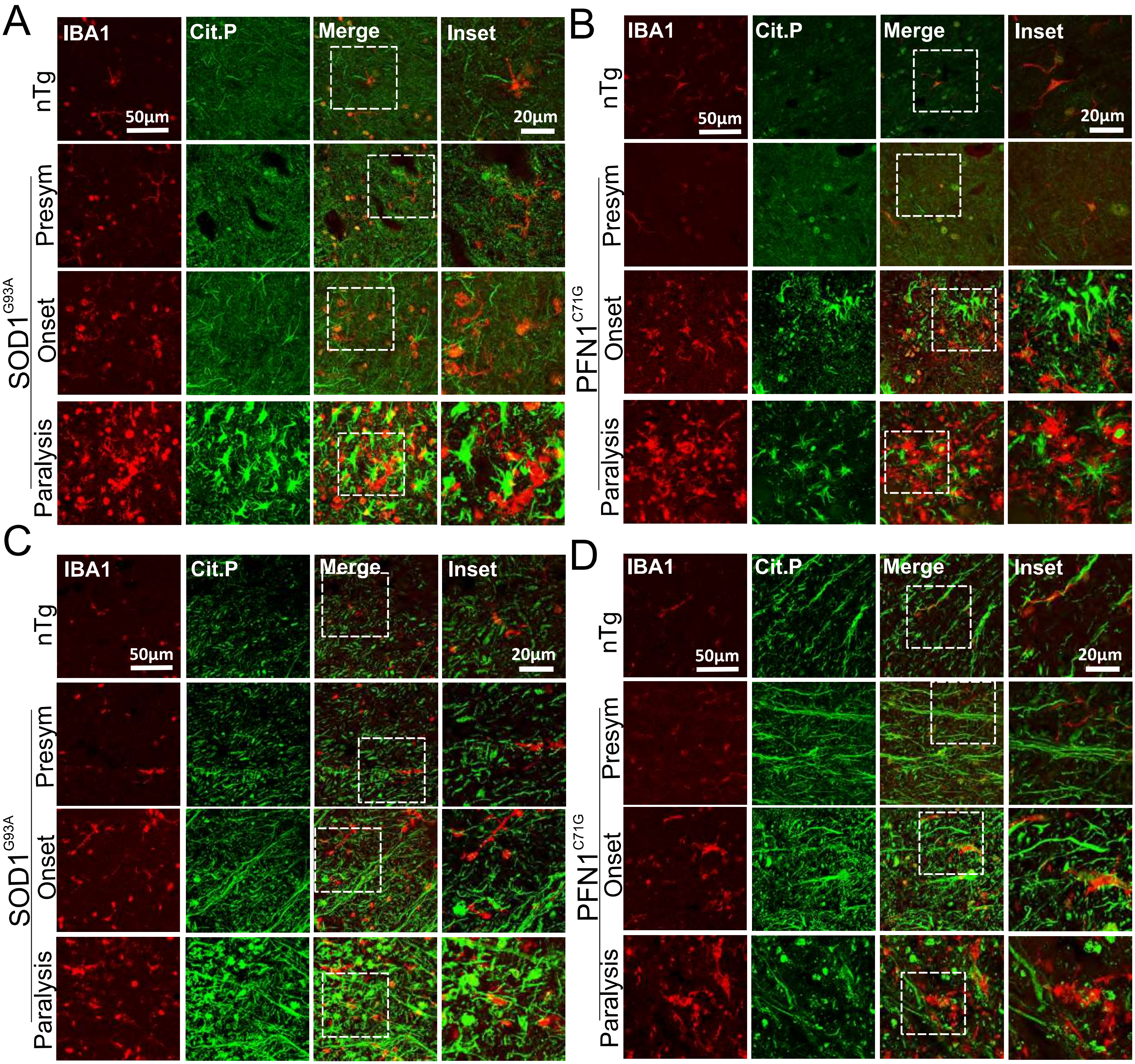

A
B
C
D
IBA1
Cit.P
Merge
Inset
nTg
Presym
Onset
Paralysis
SOD1^{G93A}
PFN1^{C71G}
50µm
20µm

Figure S3

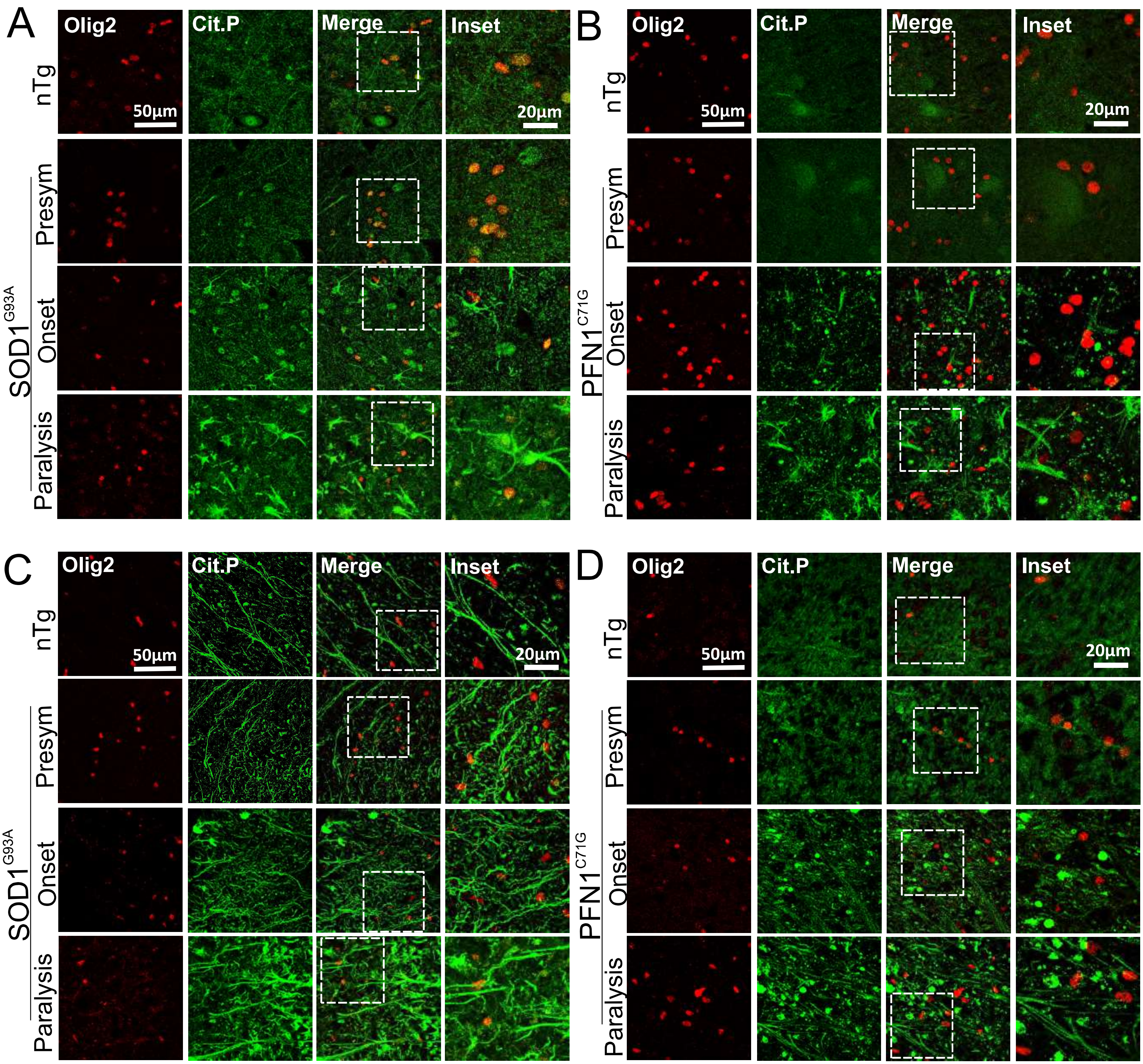
A
B
C
D
Olig2
Cit.P
Merge
Inset
nTg
Presym
Onset
Paralysis
SOD1G93A
PFN1C71G
50µm
20µm

Figure S4

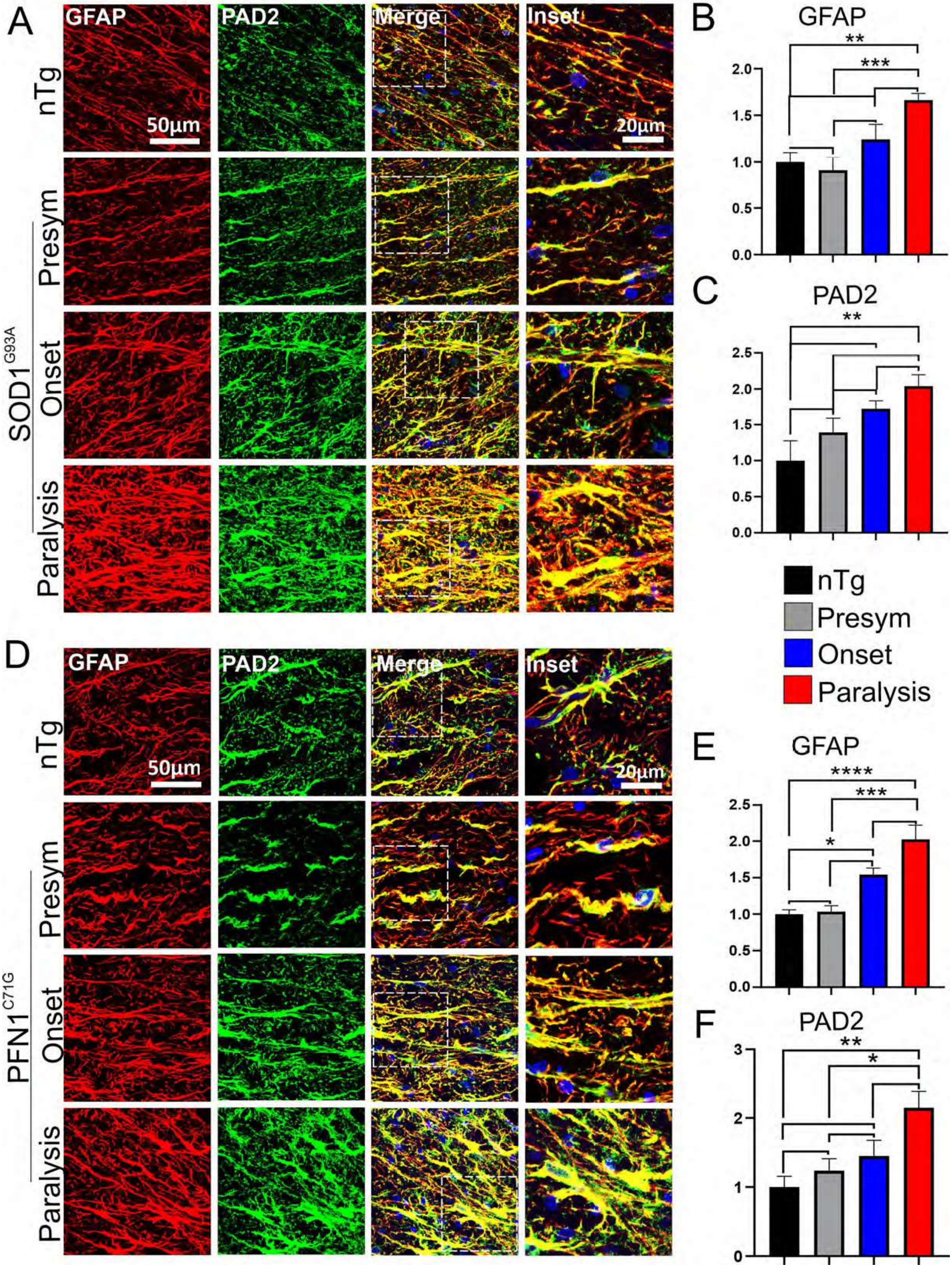

A
GFAP
PAD2
Merge
Inset
nTg
Presym
Onset
Paralysis
SOD1G93A
50µm
20µm
B
GFAP
**
***
C
PAD2
**
nTg
Presym
Onset
Paralysis
D
PFN1C71G
E
GFAP
****
***
*
F
PAD2
**
*

Figure S5

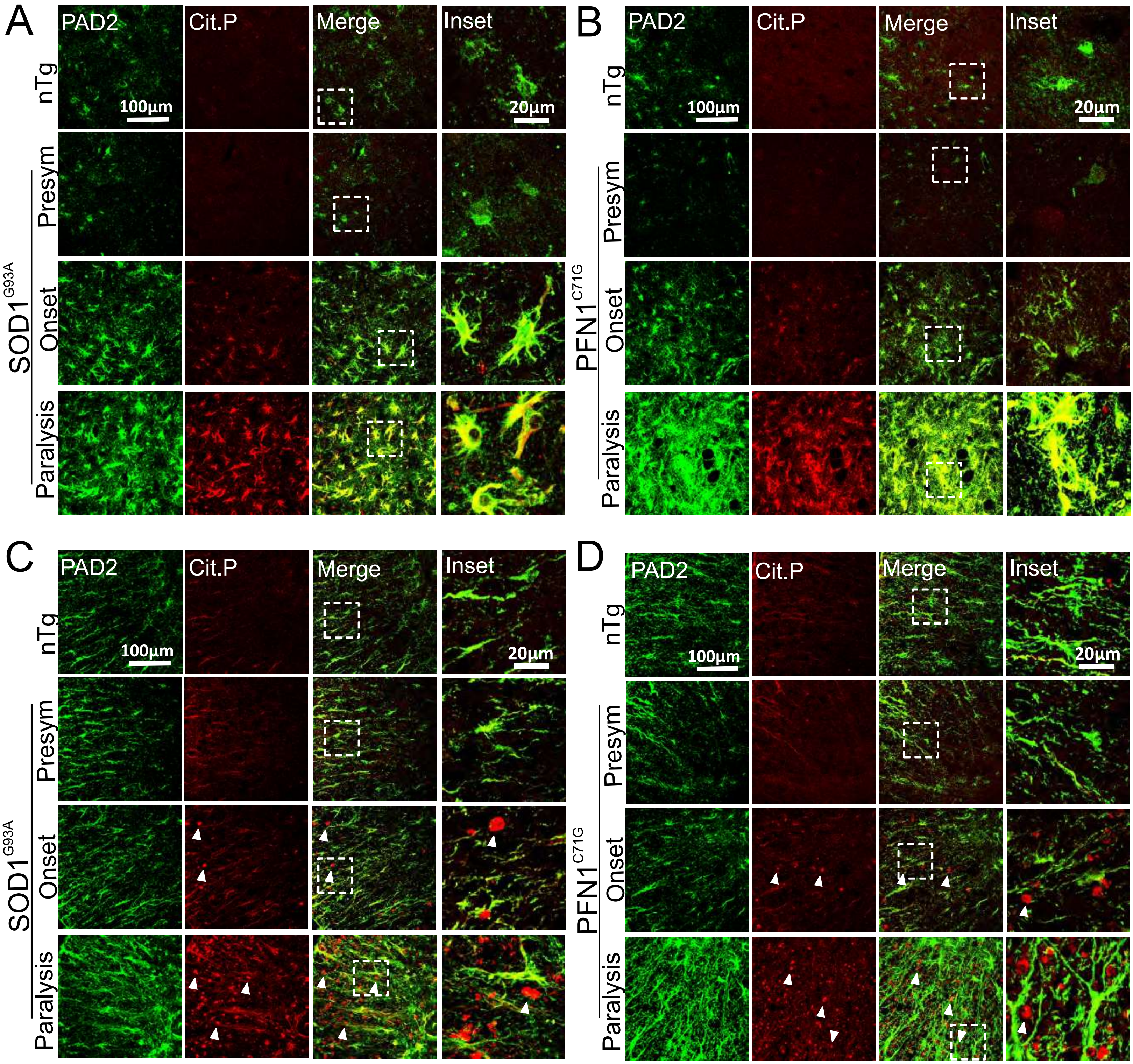

A
B
C
D
PAD2
Cit.P
Merge
Inset
nTg
Presym
Onset
Paralysis
SOD1G93A
PFN1C71G
100µm
20µm

Figure S6

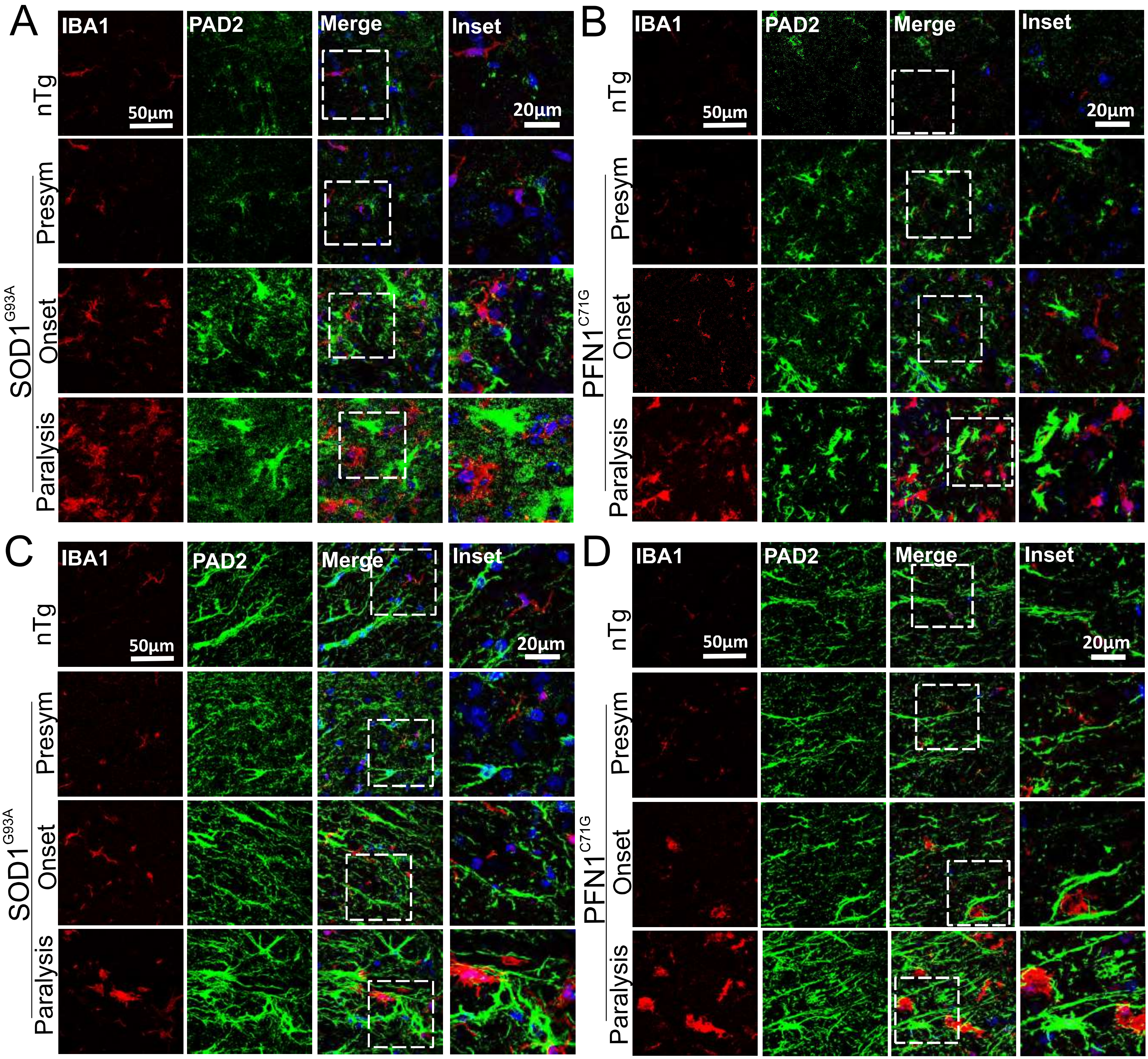

A
IBA1
PAD2
Merge
Inset
nTg
50µm
20µm
Presym
Onset
Paralysis
SOD1G93A
B
IBA1
PAD2
Merge
Inset
nTg
50µm
20µm
Presym
Onset
Paralysis
PFN1C71G
C
IBA1
PAD2
Merge
Inset
nTg
50µm
20µm
Presym
Onset
Paralysis
SOD1G93A
D
IBA1
PAD2
Merge
Inset
nTg
50µm
20µm
Presym
Onset
Paralysis
PFN1C71G

Figure S7

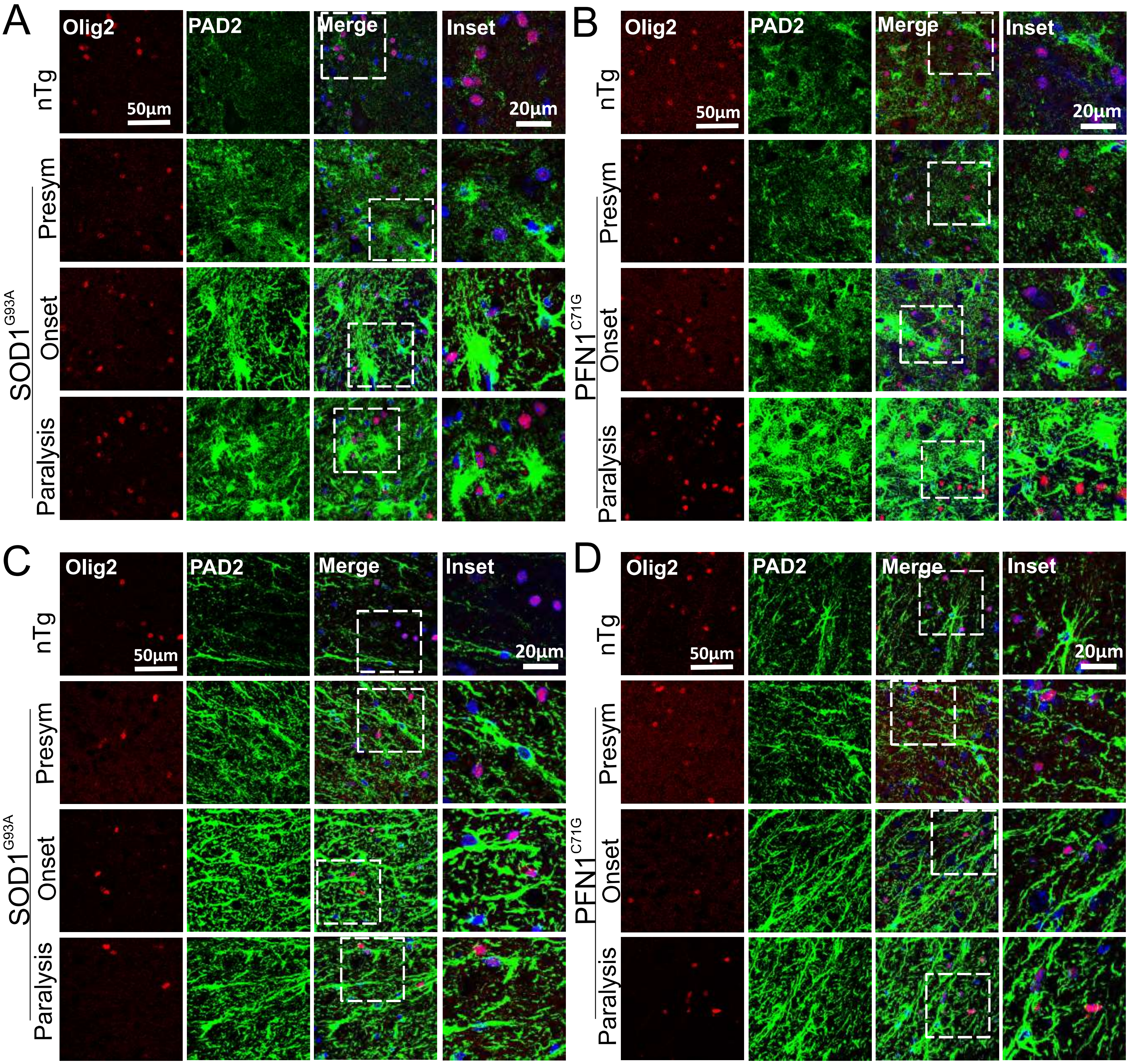
A
B
C
D
Olig2
PAD2
Merge
Inset
nTg
Presym
Onset
Paralysis
SOD1G93A
PFN1C71G
50µm
20µm

Figure S8

A

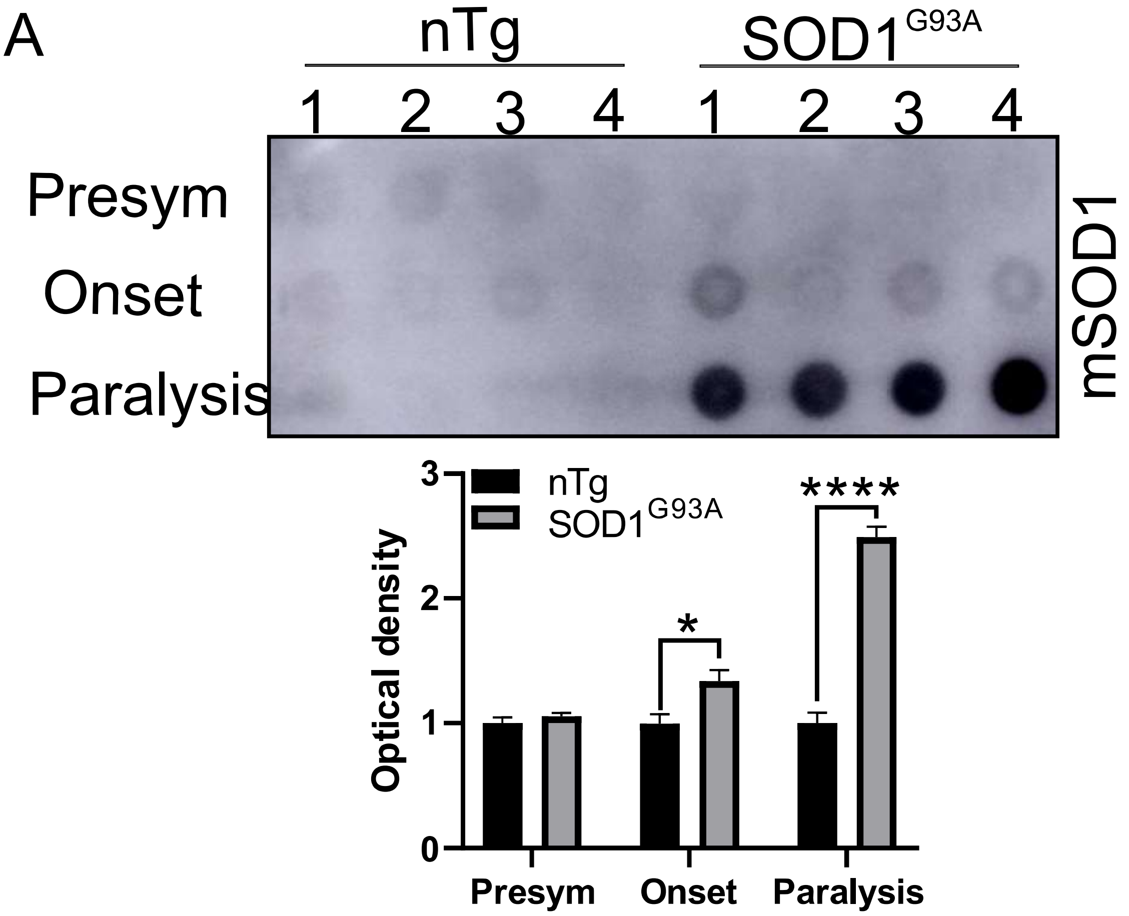

nTg
SOD1G93A
1 2 3 4 1 2 3 4
Presym
Onset
Paralysis
mSOD1
nTg
SOD1G93A
Optical density
****
*
Presym
Onset
Paralysis

B

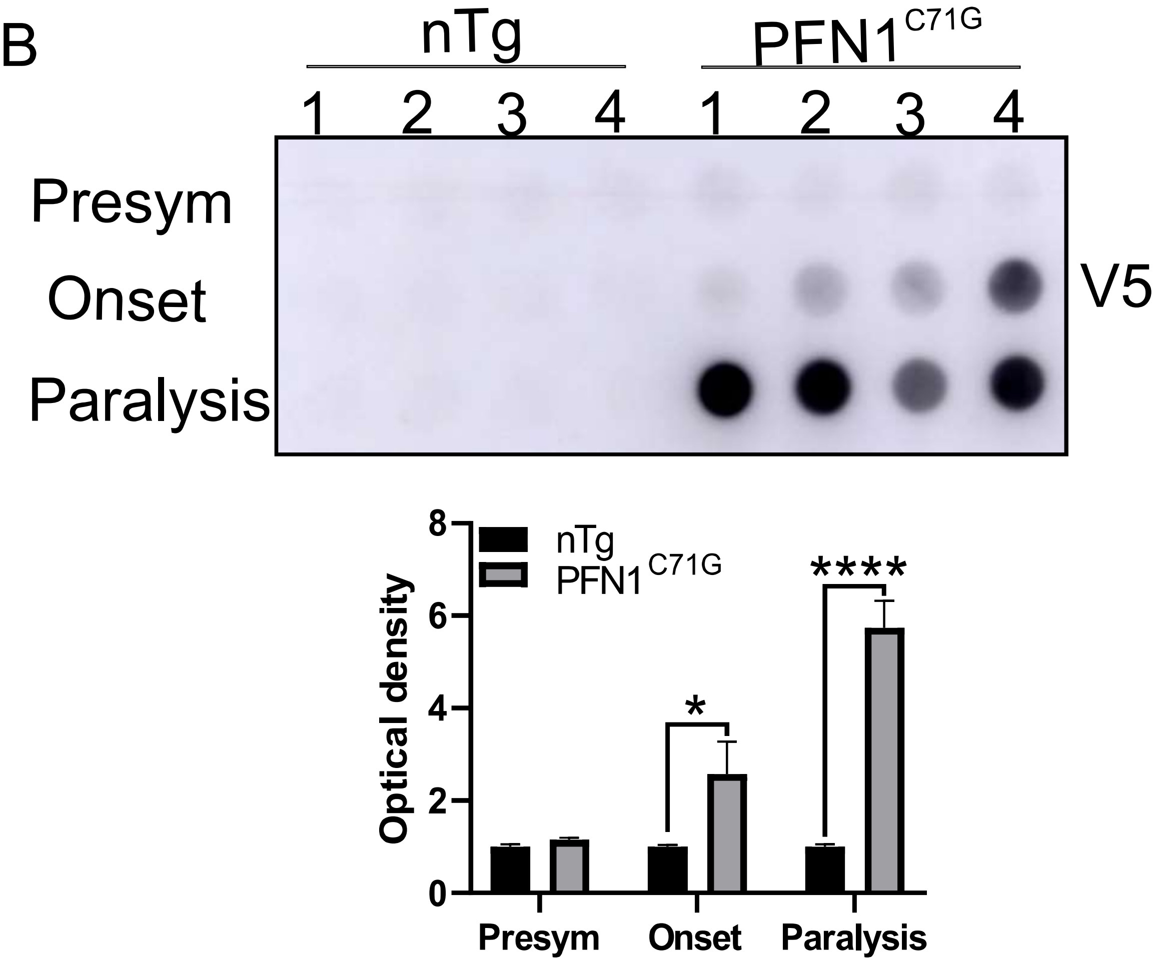

nTg
PFN1C71G
1 2 3 4 1 2 3 4
Presym
Onset
Paralysis
V5
nTg
PFN1C71G
Optical density
****
*
Presym
Onset
Paralysis

Figure S9

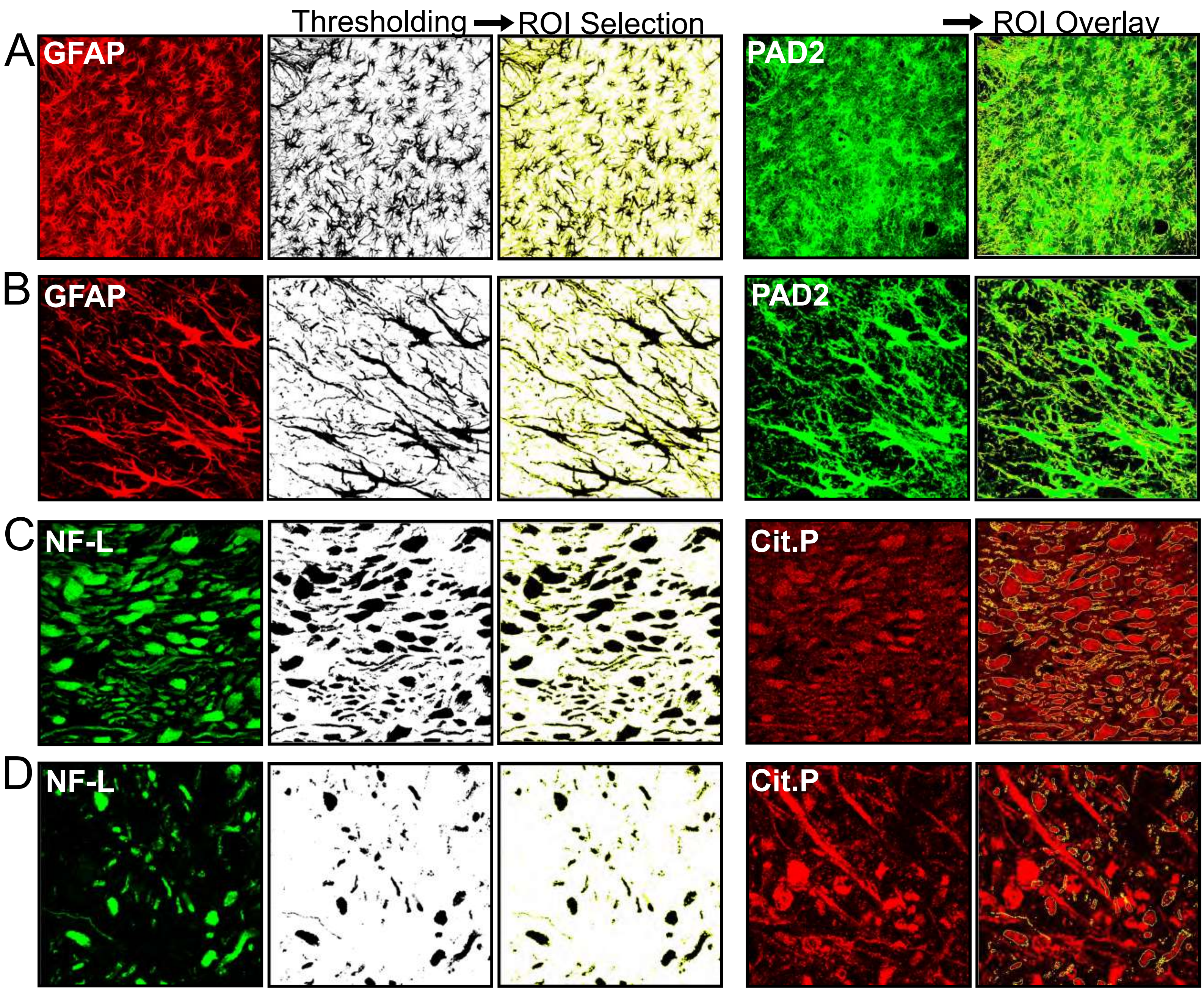
Thresholding
ROI Selection
ROI Overlay
A
GFAP
PAD2
B
GFAP
PAD2
C
NF-L
Cit.P
D
NF-L
Cit.P

Figure S10

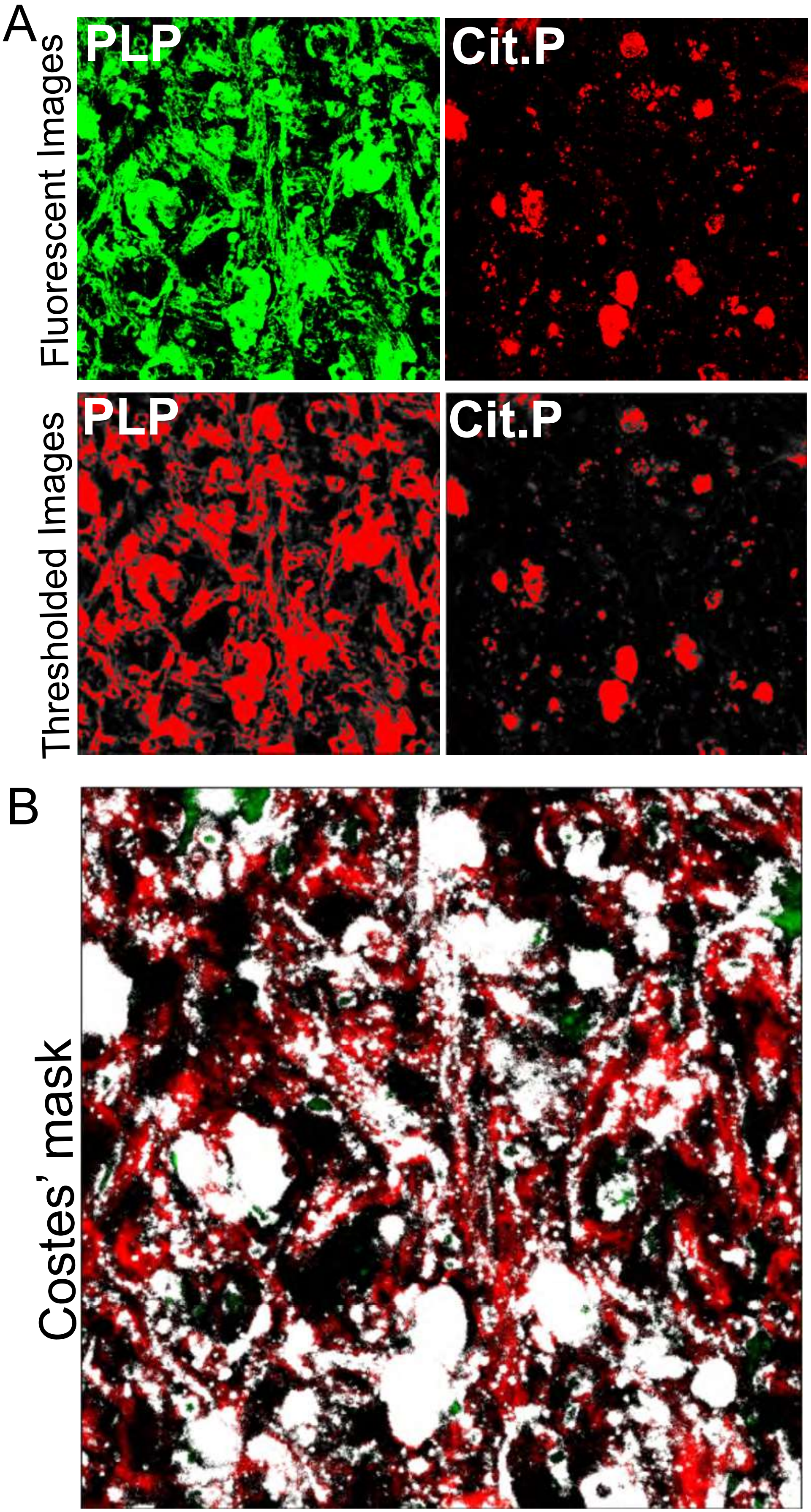
A
Fluorescent Images
Thresholded Images
PLP
Cit.P
PLP
Cit.P
B
Costes' mask
